# β-Actin Deficiency in Baraitser-Winter Syndrome Type 1 Disrupts T-Cell Function and Immune Regulation: Implications for Targeted Therapy in Actinopathies

**DOI:** 10.1007/s10875-025-01906-x

**Published:** 2025-08-01

**Authors:** Zahala Bar-On, Or Reuven, Atar Lev, Amos J. Simon, Wajeeh Salaymeh, Alit Shalom, Raz Somech, Ortal Barel, Sigal Porges, Elisheva Javasky, Vered Molho-Pessach, Zvi Granot, Dan Bijaoui, Tzahi Neuman, Yuval Tal, Michal Baniyash, Michael Berger, Oded Shamriz

**Affiliations:** 1 The Lautenberg Center for Immunology and Cancer Research, The Institute for Medical Research Israel-Canada (IMRIC), Faculty of Medicine, The Hebrew University of Jerusalem, Jerusalem, Israel; 2 Pediatric Department A and the Immunology Service, Faculty of Medicine, Jeffrey Modell Foundation Center, Edmond and Lily Safra Children’s Hospital, Sheba Medical Center, Tel Aviv University, Tel-Aviv, Israel; 3 Sheba Cancer Research Center, Institute of Hematology, Sheba Medical Center, Ramat Gan, Israel; 4 Sackler Faculty of Medicine, Tel Aviv University, Tel Aviv, Israel; 5 The Genomic Unit, Sheba Cancer Research Center, Sheba Medical Center, Ramat Gan, Israel; 6 Sheba Medical Center, Wohl Institute of Translational Medicine, Ramat Gan, Israel; 7 Department of Human Genetics, Institute for Medical Research, Hebrew University of Jerusalem, Jerusalem, Israel; 8 Department of Biotechnology, Jerusalem Multidisciplinary College, Jerusalem, Israel; 9 Department of Dermatology, Hadassah Medical Organization, Faculty of Medicine, Hebrew University of Jerusalem, Jerusalem, Israel; 10 Department of Developmental Biology and Cancer Research, Hebrew University of Jerusalem, Jerusalem, Israel; 11 Department of Pathology, Hadassah Medical Organization, Faculty of Medicine, Hebrew University of Jerusalem, Jerusalem, Israel; 12 Allergy and Clinical Immunology Unit, Department of Medicine, Hadassah Medical Organization, Faculty of Medicine, Hebrew University of Jerusalem, Jerusalem, Israel

**Keywords:** Baraitser-Winter syndrome type 1, ACTB, Immune regulation, T cells, Actinopathies, β-Actin

## Abstract

**Purpose:**

Baraitser-Winter syndrome type 1 (BRWS1) is a rare disorder characterized by intellectual disability, short stature, facial dysmorphism, cortical malformations, macrothrombocytopenia, and recurrent infections. BRWS1 is caused by loss-of-function variants in *ACTB*, leading to β-actin deficiency. Given the essential role of the actin cytoskeleton in T-cell activation, the immunological consequences of *ACTB* mutations remain unexplored. Here, we characterize immune dysfunction associated with a novel *ACTB* variant in a patient with BRWS1.

**Methods:**

Whole-exome sequencing identified a heterozygous *ACTB* p.Gln360ProfsTer4 variant in a patient with BRWS1 and combined immunodeficiency. Functional studies were performed in HEK293T cells transfected with wild-type and mutant *ACTB* constructs. Patient-derived T cells were analyzed for immunological synapse formation, cytokine production, activation, and proliferation. The therapeutic effects of exogenous IL-2 and dupilumab were evaluated.

**Results:**

The mutant β-actin protein was rapidly degraded and exerted a dominant-negative effect on wild-type β-actin, thereby disrupting cytoskeletal integrity. Patient-derived T cells demonstrated defective immunological synapse formation, reduced intra-synaptic IL-2 levels, and impaired activation and proliferation. Supplementation with exogenous IL-2 partially restored T-cell function in vitro. Notably, dupilumab treatment led to significant clinical and immunological improvement, suggesting a role in restoring immune regulation.

**Conclusion:**

BRWS1 represents a novel primary immune regulatory disorder. Our findings highlight actinopathy-driven immunodeficiency as a target for therapeutic intervention, with broader implications for cytoskeletal disorders.

**Supplementary Information:**

The online version contains supplementary material available at 10.1007/s10875-025-01906-x.

## Introduction

The T-cell cytoskeleton is a dynamic network of microtubules, actin and other filaments, which play a critical role in cellular shape, organelle trafficking, and migration [1]. In addition, T-cell polarization of actin and microtubules was previously shown to be an important step in their interactions with antigen presenting cells and immunological synapse formation, necessary for the activation, function and proliferation of these cells. Thus, cytoskeleton functionality has a direct impact on activation and proliferation of T cells [1].

Accordingly, inborn errors of immunity (IEI), where cytoskeletal function or structure is compromised, confirm its significant role in T-cell activation. For example, it was shown that deficiency in Capping Protein Regulator and Myosin 1 Linker 2 (CARMIL2) protein, which connects between vimentin and β-actin, results in abnormal T-cell migration, polarization, and activation, as well as immune dysregulation and impaired CD4^+^ T-cell differentiation [2, 3].

In addition, impaired T-cell function is observed in other actinopathies, such as dedicator of cytokinesis (DOCK)11 deficiency [4] and Wiskott-Aldrich syndrome [5]. Immune deficiency characterizing these disorders is often considered as combined and is acknowledged by the International Union of Immunological Societies (IUIS) in the updated phenotypical classification of IEI [6].

Baraitser-Winter syndrome type 1 (BRWS1) is a rare disorder affecting fewer than one in a million people. It is characterized by intellectual impairment, short stature, facial dysmorphism, cortical abnormalities, macro-thrombocytopenia and other features [7]. Genetic studies have found that BRWS1 is induced by loss-of-function variants in *actin beta (ACTB) or gamma 1 (ACTG1)* genes, which results in a deficiency of physiologically normal β- and γ-actin proteins, respectively [7, 8].

BRWS1 has been characterized by a broad spectrum of clinical symptoms [7]. Interestingly, increased susceptibility to recurrent bacterial and viral infections is a notable feature. Previously published in-vitro studies concerning BRWS1 examined neutrophils [9], fibroblasts [7], platelets [10] and natural killer (NK) cells [11]. However, studies analyzing T-cell effector functions in BRWS1 are lacking and are therefore needed.

In this study, we analyzed a patient with BRWS1 who experienced recurrent infections induced by a novel heterozygous nonsense variant in *ACTB* (p.Gln360ProfsTer4). To assess the mutation’s impact on β-actin, we overexpressed the p.Gln360ProfsTer4 variant in Human Embryonic Kidney 293 T (HEK293T) cells and found that its half-life was markedly shorter than that of wild-type (WT) β-actin. Additionally, co-expression of WT and mutant β-actin revealed that the mutant variant disrupted the normal distribution of WT β-actin, indicating a dominant-negative effect. Further investigation of the patient’s T-cells revealed defective immune synapse formation and reduced local IL-2 concentrations at the synapse. Consequently, activated T cells displayed abnormal morphology and impaired effector functions, along with a nearly complete absence of regulatory T cells (Tregs) compared to healthy controls (HC).

Based on these findings and the patient’s clinical presentation, we initiated dupilumab treatment to restore immune regulation. Remarkably, this intervention successfully partially replenished T_REGS_, reduced serum pro-inflammatory cytokines, and most importantly, led to significant clinical improvement. Overall, these results emphasize the critical impact of the *ACTB* p.Gln360ProfsTer4 mutation on T-cell functionality and the potential benefits of targeted immunotherapy in restoring immune homeostasis in patient with impaired β-actin.

## Results

### Clinical Characteristics of a Patient with Baraitser-Winter Syndrome Type 1 Reveal Immune Dysregulation and Growth Challenges

**Fig. 1:**
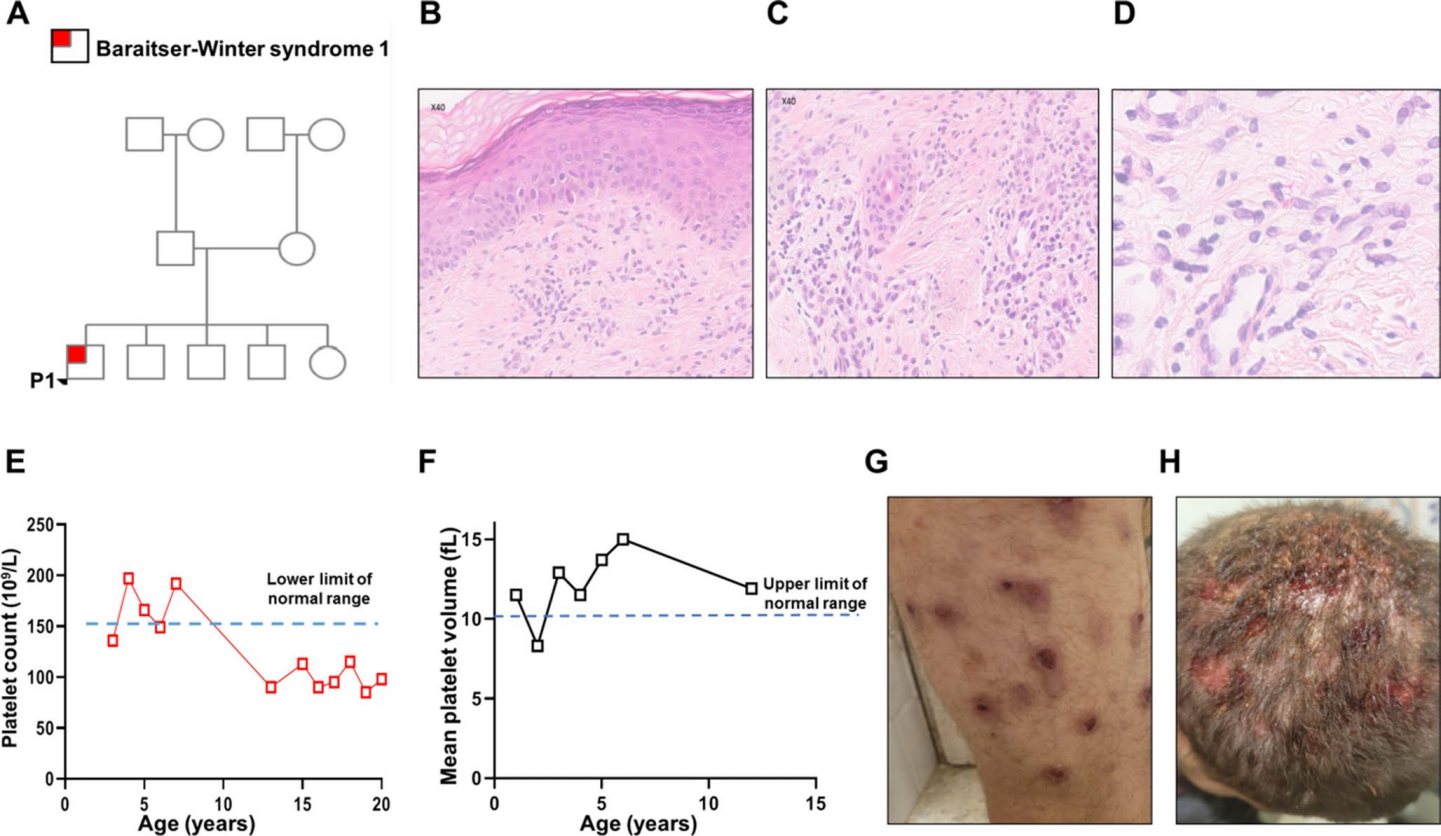
Clinical manifestations of the patient. (**A**) The family pedigree of patient (P)1 demonstrating a de-novo variant in a non-consanguineous family with 4 unaffected siblings. (**B-D**) skin biopsy pathology of P1; Punch of skin with flattened epidermis with compact hyper keratosis (**B**). In dermis- fibrosis, mild chronic inflammation including plasma cells (**C**). In the background- few eosinophils (**D**). The findings are consistent with chronic atopic dermatitis (H&E X 400). (**E**, **F**) Platelet count and mean platelet volume of P1 overtime demonstrating persistent macro-thrombocytopenia. (**G**, **H**) Severe purulent impetigo, as seen in P1’s lower limbs and scalp, respectively

Patient 1 (P1), a 20-year-old Jewish male, was treated at the Allergy and Clinical Immunology Unit at Hadassah Medical Center, Jerusalem, Israel. He was born to non-consanguineous, healthy parents and is the only symptomatic child among five healthy siblings (Fig. 1A). Physical examination revealed dysmorphic features, including hypertelorism, microcephaly, a broad nasal bridge, and coarse facial features.

The patient has chronic allergic rhinitis and severe atopic dermatitis with prurigo nodularis, which are treated with topical steroids and phototherapy (Fig. 1B-D). He also has chronic diarrhea that has not been fully evaluated or treated. His medical history includes intellectual impairment, failure to thrive, and developmental delay. Additionally, he was diagnosed with short stature characterized by a bone age delay of two years. His growth hormone levels following glucagon and arginine stimulation tests were within the normal range. Interestingly, his blood tests from early childhood revealed consistent thrombocytopenia with unusually large platelets (see Fig. 1E for platelet counts and Fig. 1 F for mean platelet volumes).

P1’s medical history is also notable for recurrent infections. He had recurrent pneumococcal pneumonia and bacteremia, including two admissions to the pediatric intensive care unit at ages 4 and 5. He also had recurrent episodes of viral acute otitis media, streptococcal periorbital cellulitis, and severe purulent impetigo (Fig. 1G, H).

His pulmonary workup included a chest computed tomography scan, which demonstrated emphysematous changes in both lung apices; diffuse thickening of air bronchi, and atelectasis of segments in the right middle and lower lobes. Spirometry revealed an obstructive disorder with a forced expiratory volume (FEV1) to forced volume capacity (FVC) ratio of 0.75. An increase in FEV1 by 14% following salbutamol treatment confirmed the diagnosis of mild asthma.

Overall, P1’s complex medical history underscores the need for a thorough evaluation and targeted management of significant immunological and developmental challenges.

### Baraitser-Winter Syndrome Type 1 in the Patient is Caused by a Novel *ACTB* Variant

**Fig. 2:**
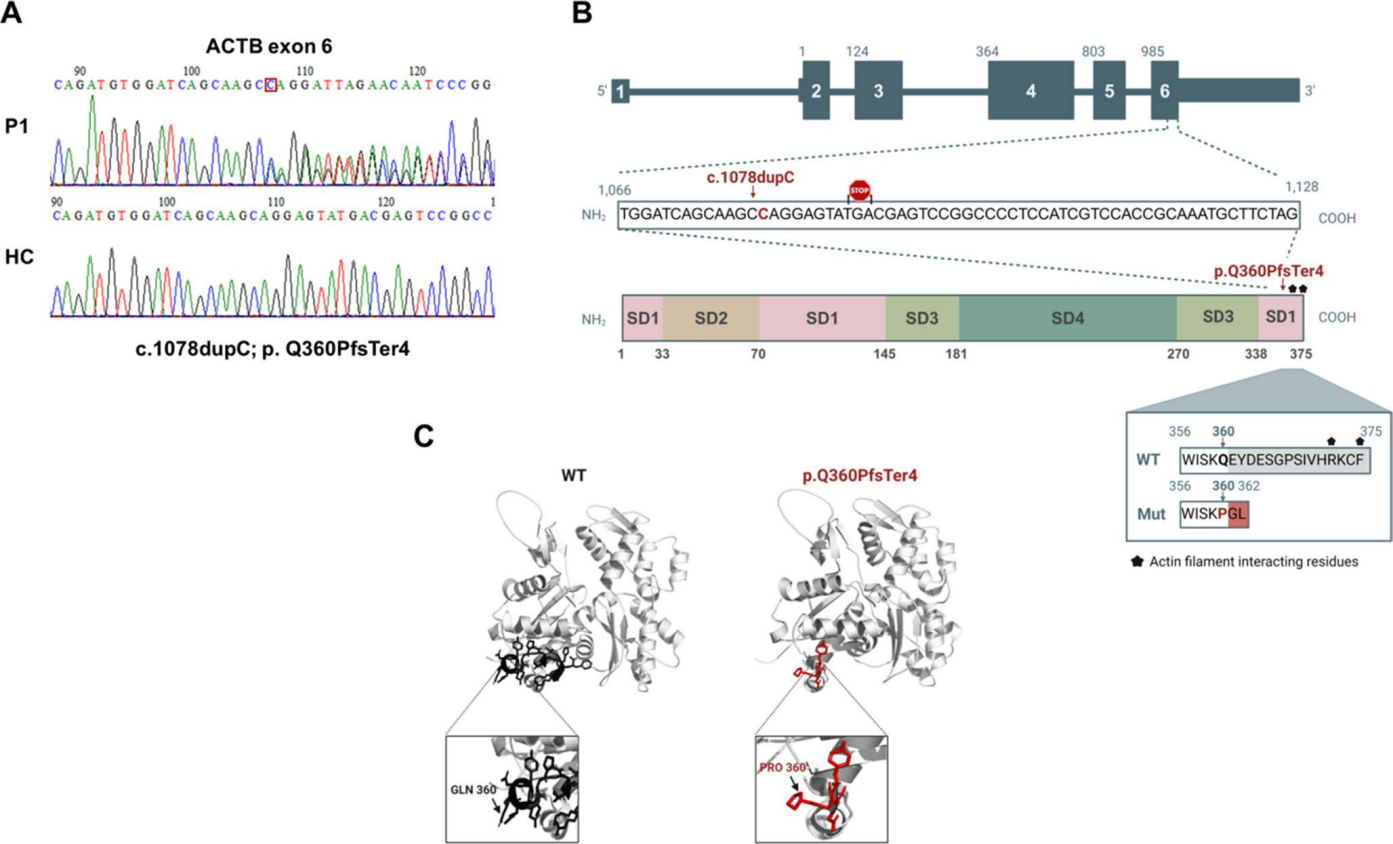
ACTB variant found in the patient. (**A**) Sanger sequencing of P1 and a healthy control demonstrates a heterozygous c.1078dupC, p.Gln360ProfsTer4 nonsense variant in Exon 6 of *ACTB*. (**B**) Position of the variant leading to premature stop codon and truncated β-actin protein (**C**) The cryo- electron microscopy model of β-actin in the patient and a HC was compiled using the PyMOL Molecular Graphics System, Version 3.0, Schrödinger, LLC. (protein data bank: 5JLH [49])

Next, using exome sequencing we identified a cytidine duplication at position 1078 in exon 6 of the *ACTB* gene, resulting in a novel heterozygous variant, c.1078dupC, p.Gln360ProfsTer4 (Fig. 2 A). This duplication leads to a frameshift that introduces a premature stop codon downstream (Ter 4), resulting in a truncated β-actin protein consisting of 362 amino acids, compared to the full-length protein of 375 amino acids (Fig. 2B). The p.Gln360ProfsTer4 variant is novel and absent from local or global databases (seenB4 score of 0). It is classified as likely pathogenic by different prediction softwares using the American College of Medical Genetics (ACMG) guidelines. Family segregation studies via Sanger sequencing revealed that neither parent of P1 harbored the *ACTB* variant, therefore characterizing it as de-novo. A cryo-electron microscopy model of β-actin in the patient and a healthy control (HC) is presented in Fig. 2C. Analysis of the truncated β-actin reveals alterations in the C-terminal domain, a critical component in actin filament dynamics and protein-protein interactions. These structural changes in the truncated β-actin may disrupt actin polymerization, stability, or binding to regulatory proteins, thus indicating possible pathogenic characteristics of the *ACTB* variant.

### The p.Gln360ProfsTer4 β-Actin Subject to Enhanced Cellular Degradation

**Fig. 3:**
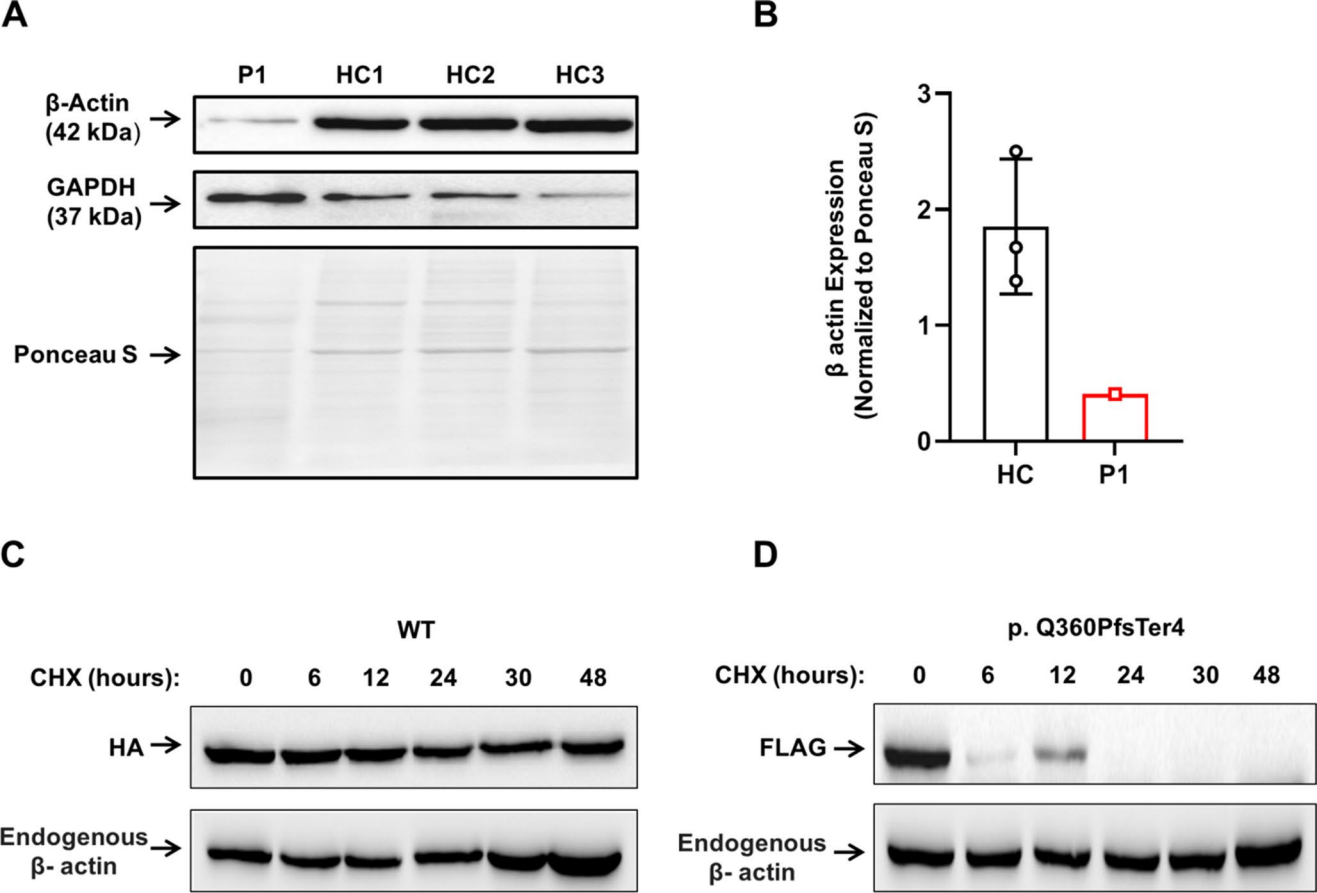
The p.Gln360ProfsTer4 β-actin protein expression and stability. (**A**, **B**) PBMC were lysed, protein extracts were separated by SDS-PAGE, transferred into a nitrocellulose membrane and tested for β-actin. GAPDH was used as a loading control. Immunoblotting has revealed that although GAPDH was increased in P1 vs. HC, expression of β-actin was nearly absent in the patient. (**C**, **D**) Human embryonic kidney 293 T (HEK293T) cells were transfected with plasmids encoding HA-β-actin (WT) and FLAG-β-actin (p.Gln360ProfsTer4 variant). Forty-eight hours post-transfection, cells were treated with cycloheximide and harvested at 0, 6, 12, 24, 30, and 48 h following treatment. The levels of exogenous WT and mutant β-actin were assessed for each time point by immunoblotting

We then sought to evaluate the pathogenicity of the p.Gln360ProfsTer4 ACTB variant by comparing the overall β-actin protein expression levels in protein extracts of peripheral blood mononuclear cells (PBMCs) from P1 and HC using immunoblotting. This analysis revealed a marked decreased β-actin expression in P1 compared to HCs (Fig. 3 A,B).

The p.Gln360ProfsTer4 variant likely affects residues 360 and beyond, including regions involve in actin polymerization (Fig. 3B) [12]. Additionally, the anti-β-actin antibody used for immunoblotting targets the same N-terminal amino acid sequence in β-actin proteins from both HC and P1 [13]. Therefore, the decrease in the β-actin band in P1’s immunoblotting may suggest enhanced degradation of the mutant β-actin. To test this, we compared the half-lives of the mutant and WT β-actin. HEK293T cells were transfected with expression plasmids for HA-β-actin (WT) and FLAG-β-actin (p.Gln360ProfsTer4 variant). Forty-eight hours post-transfection, the cells were treated with the potent eukaryotic translation elongation inhibitor cycloheximide (CHX) [14] and harvested at 0, 6, 12, 24, 30, and 48 h after treatment. We then determined by immunoblotting the levels of exogenous WT or mutant β-actin, as well as total β-actin, using antibodies against FLAG, HA, and β-actin, respectively. The expression of mutant β-actin was diminished by six hours post-CHX treatment, while the levels of WT β-actin and endogenous β-actin remained stable for up to 48 h (Fig. 3C, D).

**Fig. 4:**
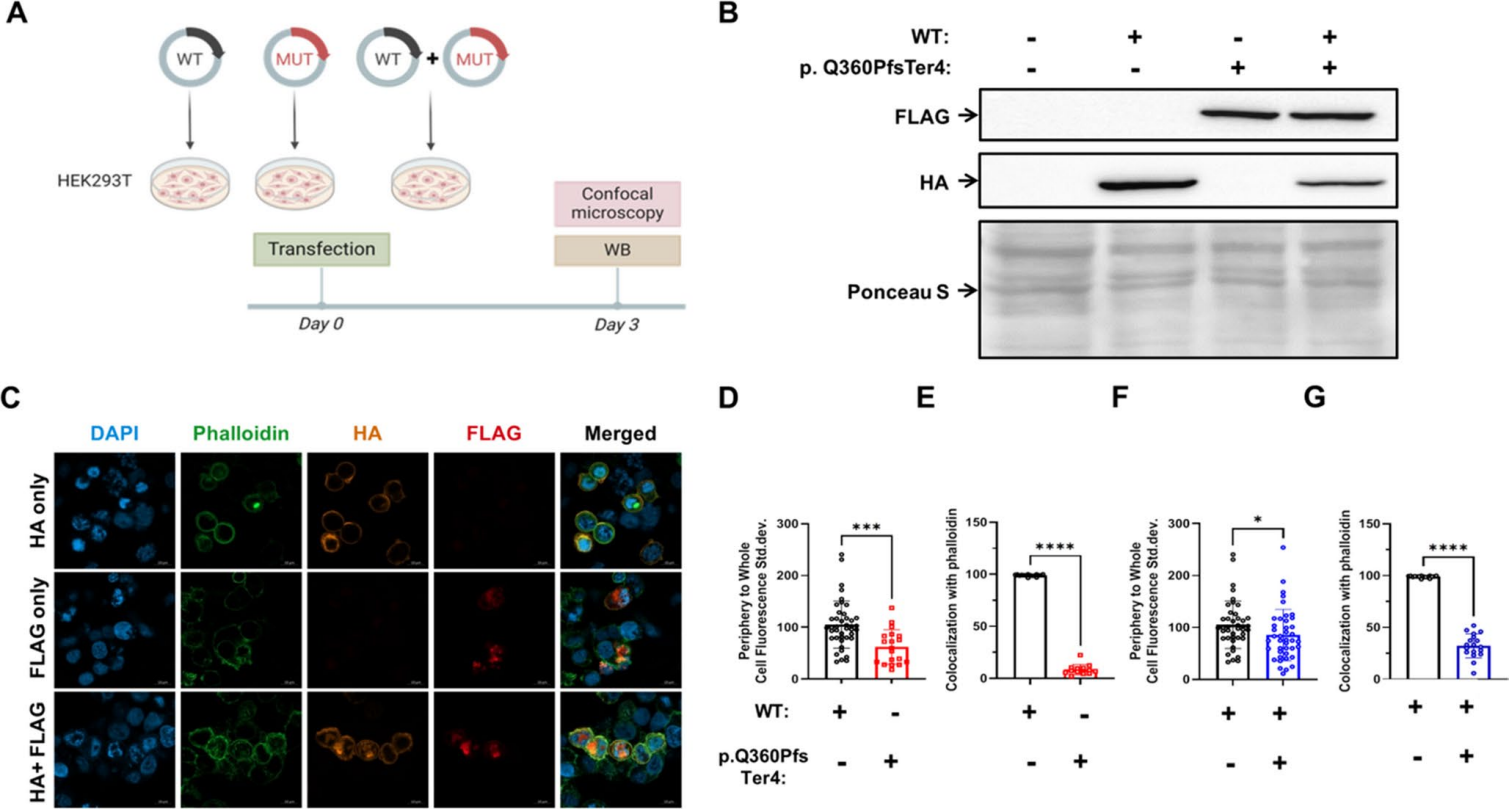
Impaired cytoskeletal localization of mutant β-actin (p.Gln360ProfsTer4) and its dominant-negative effect. (**A**) Human embryonic kidney 293 T (HEK293T) cells were transfected with expression plasmids encoding HA-tagged wild-type (WT) β-actin, FLAG-tagged mutant β-actin (p.Gln360ProfsTer4), or both. The cells were than processed to complete immunoblotting for transfection conformation and confocal microscopy of HA and FLAG. (**B**) Immunoblotting of HA and FLAG demonstrating successful transfection of WT plasmid, mutant plasmid and both. (**C**-**D**) Confocal microscopy analysis of actin cytoskeleton organization. Cells were stained with DAPI (nuclei, blue), phalloidin-iFluor 488 (endogenous β-actin, green), and antibodies against HA (WT exogenous β-actin, red) and FLAG (mutant exogenous β-actin, magenta). Periphery-to-whole-cell fluorescence standard deviation distribution was quantified in FLAG- and HA-tagged cells, representing exogenous actin distribution in WT and mutant cells. (**E**) Quantification of co-localization between exogenous and endogenous β-actin. (**F**-**G**) Periphery-to-whole-cell fluorescence standard deviation ratio and co-localization with endogenous β-actin in HEK293T cells co-transfected of WT and mutant β-actin. Each dot represents an individual cell. The two-tailed, unpaired Mann–Whitney test was used for statistical analysis. Results are presented as mean ± SD (P values: ns = non-significant; *P ≤ 0.05, **P ≤ 0.01, ***P ≤ 0.001, ****P ≤ 0.0001)

These results indicate that the half-life of the p.Gln360ProfsTer4 variant is significantly shorter than that of WT β-actin, which may explain the reduced expression observed in PBMCs from P1 (Fig. 4A, B).

### Validating the Dominant-Negative Effect of the *ACTB* Variant via In-Vitro Transfection Model

To further investigate the pathogenicity of the p.Gln360ProfsTer4 β-actin variant, we examined its effect on the actin cytoskeleton. HEK293T cells were transfected with expression plasmids for HA-tagged WT β-actin, FLAG-tagged mutant β-actin (p.Gln360ProfsTer4), or both (Fig. 4 A). Successful transfection was confirmed by immunoblotting (Fig. 4B). Confocal microscopy was used to analyze the distribution of exogenous and endogenous β-actin. Cells were stained with DAPI (nuclei), phalloidin-iFluor 488 (endogenous β-actin), and antibodies against HA and FLAG (exogenous β-actin). Transfection with the FLAG-tagged mutant β-actin plasmid resulted in a significantly reduced periphery to whole-cell fluorescence ratio compared to the HA-tagged WT β-actin plasmid (Fig. 4 C, D*;* middle vs. upper panels). Consistently, while approximately 100% of the exogenous WT β-actin co-localized with endogenous β-actin, only approximately 10% of the mutant β-actin did so (Fig. 4E). This suggests impaired cellular localization and polymerization of the mutant β-actin. Furthermore, co-transfection of WT and mutant β-actin resulted in a decreased periphery to whole-cell fluorescence ratio for the WT β-actin (Fig. 4 F) and reduced co-localization with endogenous β-actin compared to WT-only transfected cells (Fig. 4G). These findings indicate a dominant-negative effect of the p.Gln360ProfsTer4 *ACTB* variant on WT β-actin function.

### Impaired Immunological Synapse Formation and Reduced Intra-Synaptic IL-2 Concentration in T Cells with the *ACTB* Variant

**Fig. 5:**
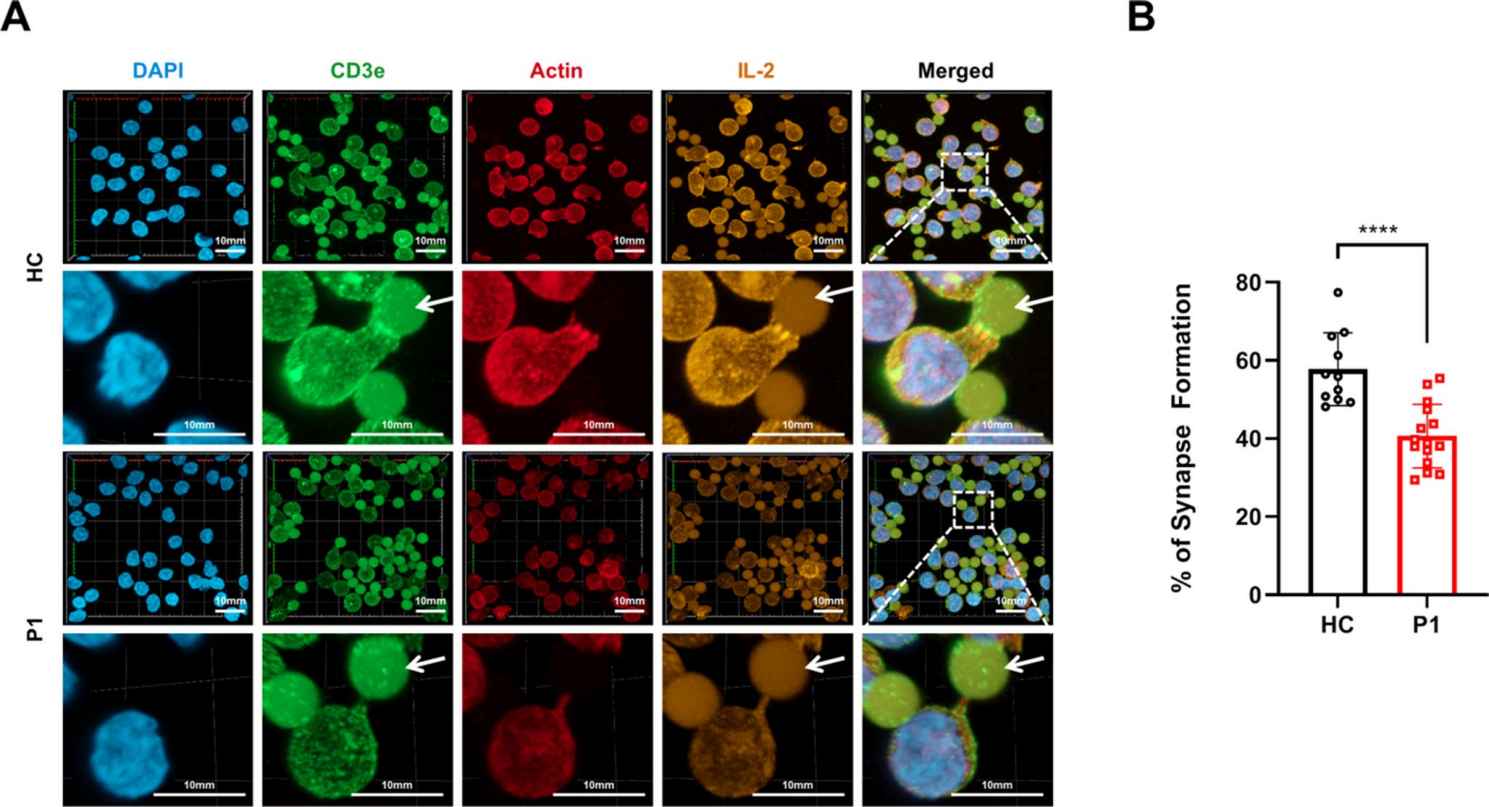
Analysis of immunological synapse formation and IL-2 localization in T cells. (**A**) CD3^+^ T cells were isolated and activated for 16 h using Dynabeads™ Human T-Expander CD3/CD28. Cells were then stained with anti-CD3 (T-cell receptor complex), Phalloidin (F-actin), DAPI (nuclei), and anti-IL-2. Confocal microscopy was used to visualize the immunological synapse and assess IL-2 distribution. Comparisons were made between T cells from the patient (P1) and a healthy control (HC). (**B**) Quantification of percentages of synapse formation out of all T-cell-beads interactions. CD28/CD3-coated activating beads are marked with white arrows. Presented is a comparison between the patient and 3 healthy controls. The two-tailed, unpaired Mann–Whitney test was used for statistical analysis. Results are presented as mean ± SD (****P ≤ 0.0001)

β-actin, a key component of the actin cytoskeleton, plays a crucial role in T-cell activation by undergoing significant rearrangement. It contributes to the formation of the immune synapse and the molecular events during T-cell activation, ultimately impacting the cell’s ability to respond to antigenic stimuli and perform its immune functions [1]. Therefore, we hypothesized that T-cell activation and function in P1 are impaired. We first examined the formation of immunological synapses following activation. CD3^+^ T cells were isolated and activated for 16 h using Dynabeads™ Human T-Expander CD3/CD28. The T cells were then stained for CD3, actin, IL-2 and nucleus. Confocal microscopy analysis revealed defective immunological synapse formation upon activation in P1 as compared to HC, with reduced local intra-synaptic IL-2 concentrations in the patient (Fig. 5A, B). Noteworthy, the TCRα,β and CD28 expression in T cells from P1 and HCs was comparable (Supplementary Fig. 1). These findings suggest that T cells carrying the *ACTB* variant fail to form immunological synapses with antigen-presenting cells properly. Moreover, the reduced intra-synaptic IL-2 concentrations indicate impairment in signal transduction required for effective T-cell activation.

### The Dominant-Negative *ACTB* Variant Disrupts Activated T-Cell Morphology

As immunological synapse formation and intra-synaptic IL-2 concentration were impaired in T-cell from P1, we next aimed to evaluate T-cell activation of P1 and to assess the effect of exogenous IL-2. Our group previously reported that activation and proliferation of CARMIL2-deficient T-cell could be rescued in-vitro by adding exogenous IL-2 [15]. Similarities between CARMIL2 deficiency and *ACTB*-related BRWS1 in P1, including T-cell cytoskeleton dysfunction and immune dysregulation, prompted us to attempt rescuing P1’s impaired T-cell effector functions by adding different doses of exogenous IL-2 to the activation medium.

**Fig. 6:**
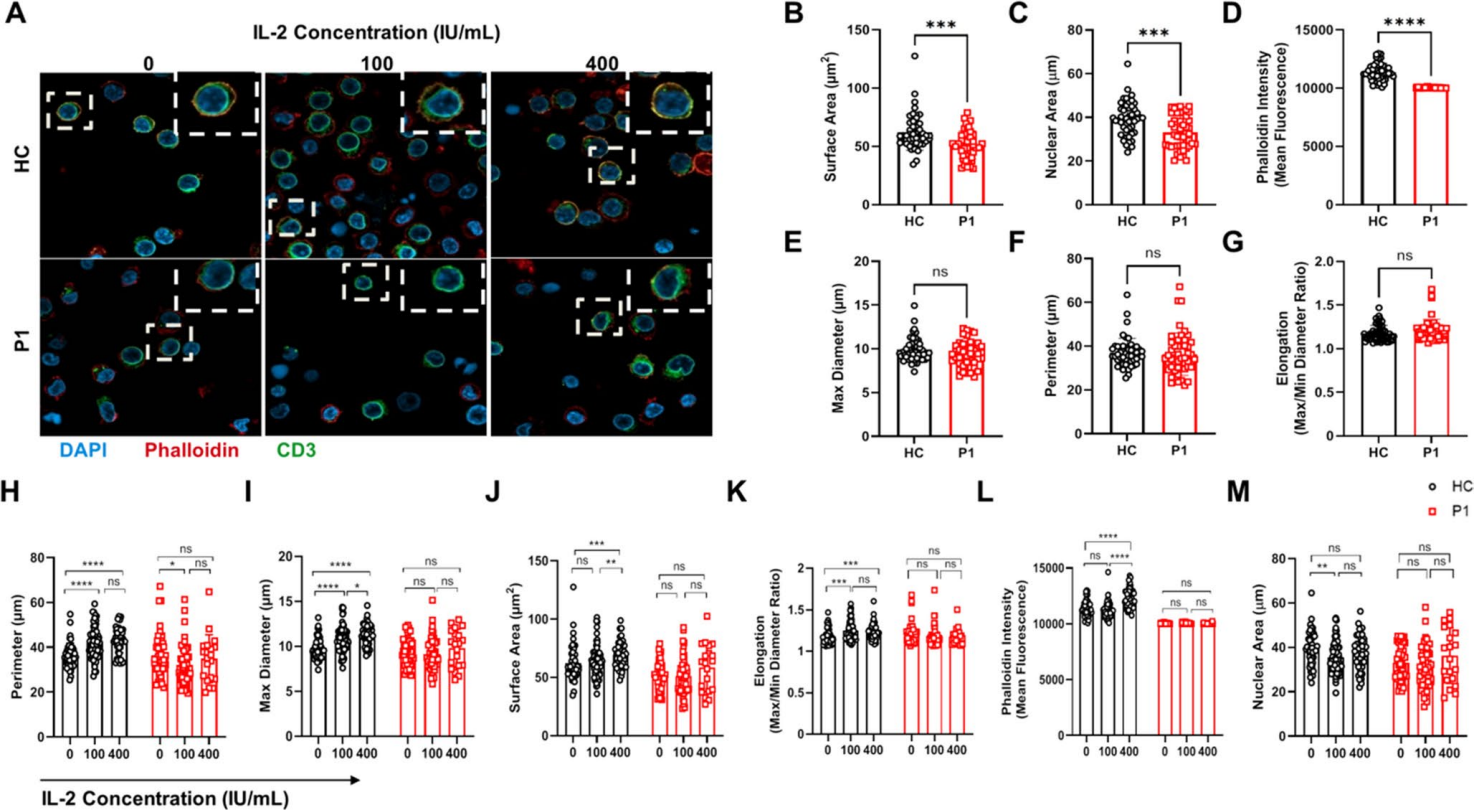
Morphological analysis of activated T cells and the effect of exogenous IL-2. (**A**) PBMCs from the patient (P1) and healthy controls (HC) were activated for 24 h with or without the addition of exogenous IL-2. Cells were stained with phalloidin (F-actin), DAPI (nuclei), and anti-CD3 antibody to assess T-cell morphology. Confocal microscopy was used to visualize and quantify cellular and nuclear morphology. (**B-G**) Several morphological parameters were analyzed, including surface area, nuclear area, β-actin levels, maximal diameter, cellular perimeter, and cellular elongation. (**H-M**) The effect of exogenous IL-2 on these parameters was assessed in both HC and P1 T cells. Each dot represents an individual cell. The two-tailed, unpaired Mann–Whitney test was used for statistical analysis. Results are presented as mean ± SD (P values: ns = non-significant; *P ≤ 0.05, **P ≤ 0.01, ***P ≤ 0.001, ****P ≤ 0.0001)

Upon activation, T cells undergo a marked increase in size within the first 24 h [16], accompanied by extensive cytoskeletal remodeling [17], including actin polymerization [18] and elongation-flattening of the activated cells [19]. Additionally, during activation, T cells increase their nuclear size through chromatin de-condensation, which facilitates the upregulation of activation-related proteins [20]. To investigate these early events in T-cell response to activation, PBMCs from P1 and HC were activated for 24 h with or without the addition of exogenous IL-2 at different concentrations. After 24 h of activation, the cells were stained for actin (using phalloidin), CD3 and nucleus, and then visualized using confocal microscopy (Fig. 6A). The subsequent analysis focused on several morphological parameters, including surface area, maximal diameter, cellular perimeter, cellular elongation (the ratio of maximal to minimal diameter) and nuclear area of the activated T cells. Phalloidin signal intensity was also measured to quantify T-cell β-actin levels. In comparison to WT, P1 T cells demonstrated a significant reduction in surface area (Fig. 6B), nuclear area (Fig. 6 C), and β-actin levels (Fig. 6D). No significant alterations were observed between the two groups in maximal diameter (Fig. 6E), cellular perimeter (Fig. 6 F), and cellular elongation (Fig. 6G). These results indicate abnormal cell morphology and confirm β-actin deficiency in P1, consistent with the immunoblotting results (Fig. 3D). Furthermore, while exogenous IL-2 increased all these morphological parameters in a dose-dependent manner in HC’s activated T cells, it had no effect on the T cells carrying the p.Gln360ProfsTer4 *ACTB* variant (Fig. 6 H-L). However, there was no evidence that exogenous IL-2 increased the nuclear area in either group (Fig. 6M).

### Impaired Activation and Proliferation of T Cells with the *ACTB* Variant, Partially Rescued In-Vitro by Exogenous IL-2

**Fig. 7:**
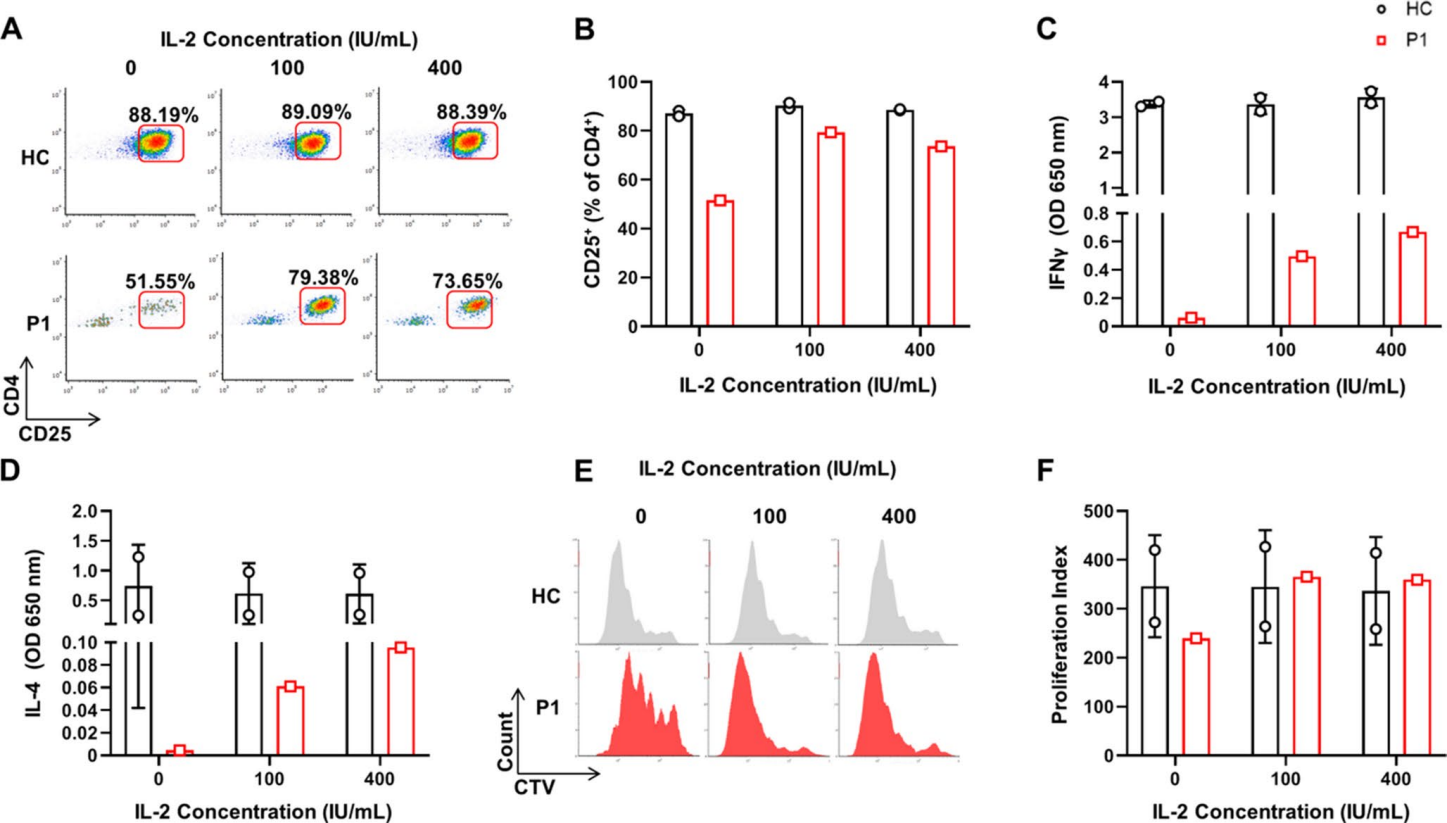
Analysis of T-cell activation, cytokine secretion, and proliferation in the presence of exogenous IL-2. (**A**-**B**) PBMCs from the patient and a healthy control were activated in vitro for 48 h, followed by flow cytometry analysis of CD25 (IL-2 receptor α) expression in CD4^+^ effector T cells. (**C**-**D**) Interferon (IFN)γ and IL-4 cytokine levels were measured in the supernatants of 5-day-activated T cells to assess Th1 and Th2 responses. Quantification of cytokines was conducted by ELISA (**E**-**F**) T-cell proliferation capacity was evaluated using CellTrace Violet (CTV) staining and flow cytometry. Presented are histograms of CTV staining. The effect of different doses of exogenous IL-2 on these parameters was also examined

Due to the lack of impact of exogenous IL-2 on morphological parameters of the patient’s T cells, we next aimed to examine its effect on effector functions. PBMC from the HC and the patient were activated in-vitro. After 48 h of activation, light microscopy revealed that T cells from P1 exhibited impaired formation of active T-cell rosettes, unlike those from HC (Supplementary Fig. 2). We then analyzed different T-cell effector functions using flow cytometry. Upregulation of CD25 (α subunit of IL-2 receptor) was reduced in P1’s effector CD4^+^ T cells compared to those of the HC (Fig. 7 A, B). Additionally, IFN-γ and IL-4 levels in the supernatants of 5-day-activated T cells were markedly decreased in P1 compared to the HC, indicating impaired Th1 and Th2 cytokine secretion, respectively (Fig. 7 C, D). Lastly, CD4^+^ T-cell proliferation capacity was also impaired, as demonstrated by CellTrace Violet (CTV) staining and flow-cytometry readout (Fig. 7E, F). Overall, these results demonstrated decreased activation and proliferation of CD4^+^ T cells in P1 with the dominant negative *ACTB* variant. Interestingly, a partial rescue was noted at higher IL-2 doses, as evidenced by CD25 upregulation (Fig. 7A-B), IFNγ and IL-4 secretion during activation (Fig. 7C-D), and CD4^+^ T-cell proliferation capacity (Fig. 7E-F). Measurements of secreted IL-4 and IFNγ following 5 days of activation revealed that the rescue of activation by addition of exogenous IL-2 was dose-dependent (Fig. 7C-D). Similar findings regarding decreased effector function and IL-2 partial rescue were observed for CD8^+^ T cells (Supplementary Fig. 3A-D). These findings are notably similar to our previous report demonstrating the rescue of activated CARMIL2-deficient T cells by adding exogenous IL-2 in-vitro [15]. However, despite the rescue in activation and proliferation, CD4^+^ and CD8^+^ T-cell post-activation counts were significantly reduced compared to the HC (supplementary Fig. 4A-C). Correspondingly, increased dead cell counts were noted in P1 (Supplementary Fig. 4D). In summary, these results demonstrate that T cells harboring the p.Gln360ProfsTer4 *ACTB* variant are characterized by impaired cellular morphology, activation and proliferation. While addition of exogenous IL-2 partially rescued activation and proliferation, it did not significantly affect the morphology of T cells harboring the p.Gln360ProfsTer4 *ACTB* variant.

### Immune Evaluation Reveals Specific Antibody Deficiency, Immune Dysregulation with Hyper-IgE Levels, and Significantly Reduced Memory T-Cell Subsets

**Fig. 8:**
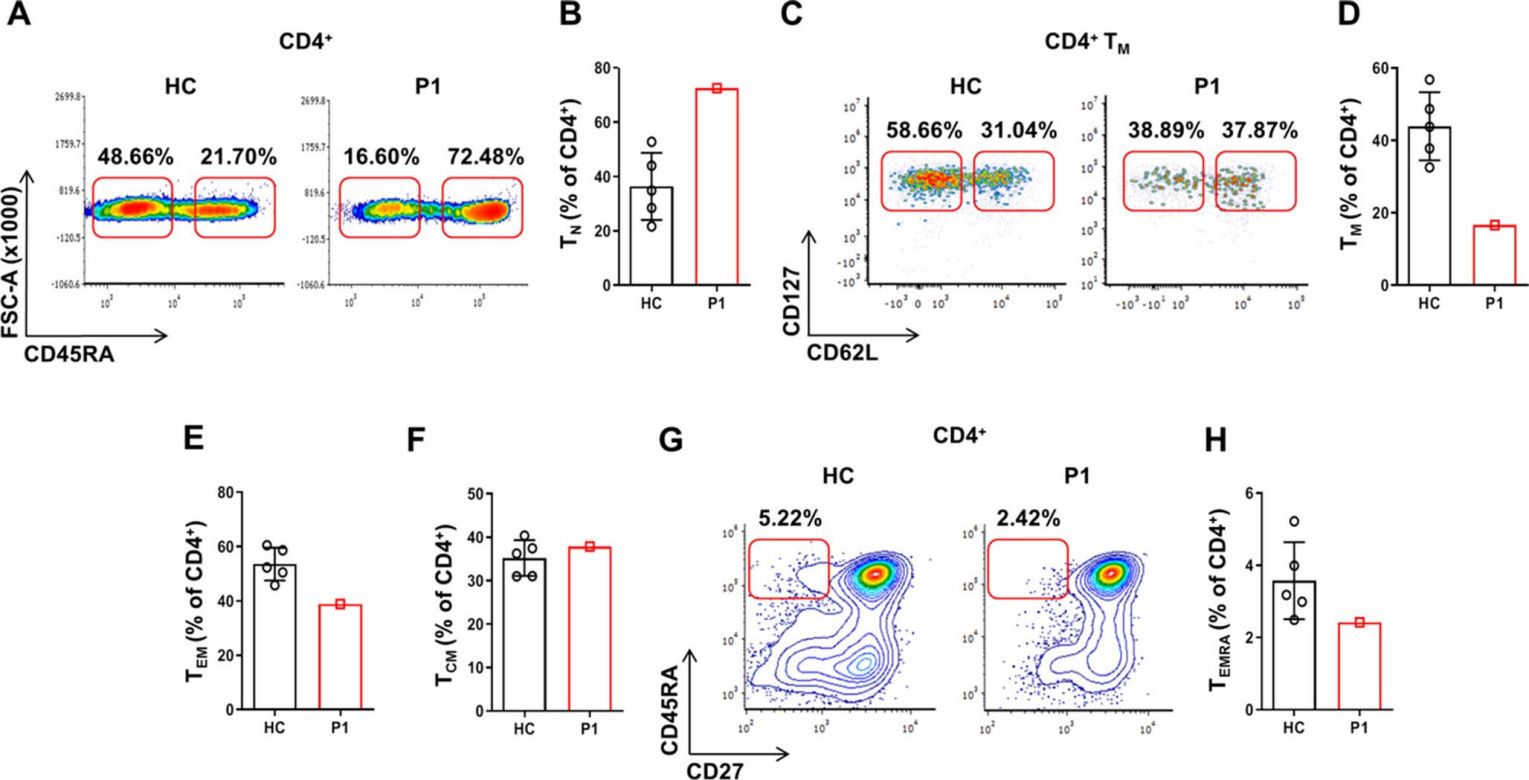
Immune phenotyping analysis of T-cell subsets in the patient. PBMCs were purified from the patent’s peripheral blood, stained for surface markers and analyzed by flow cytometry for T-cell specific subsets. Data are presented as density plots and summarized in bar graphs. (**A-F**) Flow cytometric analysis of CD4^+^ T-cell subsets, including naïve (T_N_), total memory (T_M_), effector memory (T_EM_) and central memory (T_CM_) populations. (**G-H**) As explained above, for effector memory cells re-expressing CD45RA (T_EMRA_) CD4^+^ T-cell subset

Upon admission to our clinic, an immune evaluation was initiated (Table 1). A complete blood count indicated normal levels of absolute leukocytes, lymphocytes, and eosinophils; however, absolute monocyte counts were consistently elevated, with a value of 1.2 × 10^9^/L (normal range: 0.28–0.5 × 10^9^/L). Flow cytometry analysis showed an increased quantity of the naïve T-cell subset (T_N_) with a corresponding decrease in total memory (T_M_) and effector memory (T_EM_) CD4^+^ T-cell subsets in P1, as compared to HC (Fig. 8A-E*)*. T_EMRA_ CD4^+^ population was also reduced in P1 (Fig. 8G, H). Analysis of CD8^+^ T cells demonstrated similar results (Supplementary Fig. 5). B cell and NK cell counts were within normal limits. In terms of humoral immunity, hypergammaglobulinemia was observed, with increased levels of total IgG (1955 mg/dL; normal range: 639–1349 mg/dL), as well as IgG1 and IgG4 levels. Conversely, IgM levels were low at 25 mg/dL (normal range: 40–230 mg/dL). Extremely elevated IgE levels were detected, peaking at 16,700 U/mL (normal range: 0-100 U/mL). Furthermore, IgG titers against previous vaccines—including measles, mumps, rubella, varicella, hepatitis A and B, tetanus, pertussis, diphtheria, and *Streptococcus pneumoniae*—were all negative. This indicates a specific antibody deficiency despite elevated total IgG levels; therefore, the patient was advised to initiate monthly immune globulin replacement therapy (Table 1). Along with recurrent infections, P1 displayed atopy, including atopic dermatitis, asthma and allergic rhinitis. Consistent with immune dysregulation, P1 was found to have nearly absent circulating CD4^+^CD25^+^FOXP3^+^ (forkhead box protein 3) T_REGS_ counts (0.96%; normal range: 4.2–9.9%). In addition, flow cytometry analysis of the TCR V-β repertoire revealed a normal, polyclonal distribution. However, a notable expansion of the Vβ3 clone was observed in the patient compared to HC, suggesting a potential autoreactive clonal expansion (Supplementary Table 1). Finally, since BRWS1 has been previously associated with neutrophil dysfunction [9], we aimed to assess reactive oxygen species (ROS) production in neutrophils using two methods: chemiluminescence and DHR assays. The results indicated that although P1’s neutrophils generated some ROS, both the total production and secretion in response to PMA activation were reduced in the patient compared to HC (Supplementary Fig. 6 A-B). Unfortunately, the patient declined the offered intravenous immunoglobulin and prophylactic antibiotic treatments, despite his history of recurrent infections and specific antibody deficiency. In summary, the immunological analysis of P1 indicated a phenotype of combined immune deficiency consistent with features of a primary immune regulatory disorder.

**Table 1:** Immune workup of the patient with dominant negative-*ACTB* variant

Parameter				P1 (20 yo)	Normal Range
Absolute leukocyte count (10^9^/L)				9.7	4.5–11.0
Absolute lymphocyte count (10^9^/L)				3.3	1.0-4.8
Absolute eosinophil count (10^9^/L)				0.5	0.0-0.5
Absolute monocyte count (10^9^/L)				**1.2**	0.28–0.5
Immune phenotyping	T cells	CD3^+ (^%)		62	59.3–88.6
		CD4^+^ (%)	Total	*44*	47.7–82.7
			Naïve (CD45RA^+^)	27.2	10.0–66.6
			Memory (CD45RO^+^)	*9.5*	32.3–89.0
			Tregs (CD25^+^FOXP3^+^)	*0.96*	4.2–9.9
		CD8^+^ (%)	Total	18	13.9–44.0
			Naïve (CD45RA^+^)	14.3	10.0-71.2
			Memory (CD45RO^+^)	*1.7*	20.7–70.4
		CD4^+^/CD8^+^ Ratio	2.4:1		(1.8–2.2):1
	NK cells	CD56^+^ (%)	15		4.4–18.4
	B cells	CD20^+^ (%)	15		13.0–21.0
TCR V-β repertoire				Polyclonal/normal; Mild expansion of Vβ3 clone	-
Serum Ig		IgG (mg/dL)		**1955**	639–1349
		IgA (mg/dL)		214	70–312
		IgM (mg/dL)		*25*	40–230
		IgE (U/mL)		**16,700**	3-100
IgG subtypes		IgG1 (mg/dL)		**1050**	362–1027
		IgG2 (mg/dL)		293	81–472
		IgG3 (mg/dL)		36.90	13.80-105.80
		IgG4 (mg/dL)		**375**	4.90-198.50
Specific IgG antibodies^*^		Measles (AU/mL)		*Negative*	≥ 1.1
		VZV (AI)		*Negative*	≥ 1.1
		Rubella (IU/mL)		*11.8*	> 30
		Mumps (AU/mL)		*< 5*	> 24
		HBV surface (S/CO)		*9.9*	> 15
		HAV (S/CO)		*Negative*	0
		*Bordetella Pertussis* (IU/mL)		*< 5*	> 70
		Diphtheria (IU/mL)		*< 0.01*	> 0.01
		Tetanus (IU/mL)		*0.011*	> 0.51
		*Strep. Pneumoniae* (mg/dL)		*1.6*	> 27.0

NK- Natural killer; Ig- Immunoglobulins; HBV- Hepatitis B virus; HAV- Hepatitis A virus; VZV- Varicella Zoster Virus. In **bold**- values above normal range; *Italics–* below normal range. * Exact numerical value is not available for measles, VZV and HAV IgG titers

### Dupilumab Treatment for the Patient Successfully Alleviates Chronic Inflammation

**Fig. 9:**
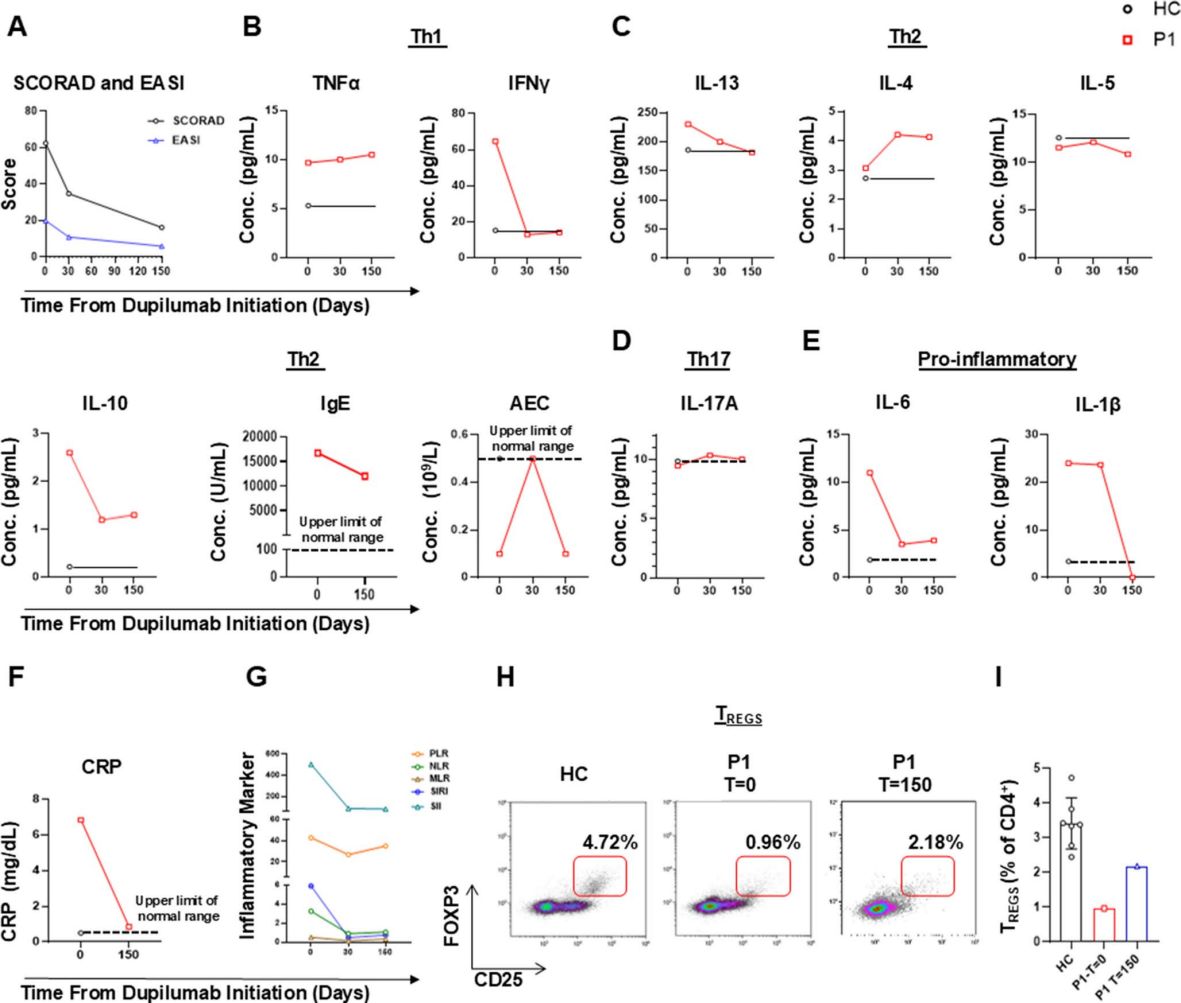
Clinical and Immunological responses to dupilumab treatment. (**A**) Eczema area and severity index (EASI) and Scoring atopic dermatitis (SCOARD) clinical scores of the patient’s atopic dermatitis during dupilumab treatment. (**B-F**) Longitudinal analysis of serum Th1, Th2, Th17 and pro-inflammatory cytokines, IgE levels, absolute eosinophil count and C-reactive protein at baseline (t = 0), and after 30 and 150 days of dupilumab treatment. (**G**) Blood count-derived inflammatory markers during dupilumab treatment, including the platelet-to-lymphocyte ratio (PLR), monocyte-to-lymphocyte ratio (MLR), neutrophil-to-lymphocyte ratio (NLR), systemic inflammation response index (SIRI), and systemic immune-inflammation index (SII). (**H-I**) Flow cytometric analysis of circulating regulatory T cells (T_REGS_) at baseline and during treatment with dupilumab

To address the patient’s immune dysregulation and symptoms consistent with type 2 inflammation, dupilumab, a monoclonal antibody targeting the IL-4/IL-13 receptor α subunit, was initiated at a dose of 300 mg every two weeks. Baseline serum cytokines were quantified in both the patient and HCs using ELISA, revealing elevated levels of Th1 cytokines (TNFα, IFN-γ), Th2 cytokines (IL-4, IL-13, IL-10), and pro-inflammatory cytokines (IL-6, IL-1β) (Fig. 9 A-E). Serum levels of IL-5 and IL-17 A were similar to those in HC, with IL-5 levels corresponding to the normal eosinophil counts observed in the patient (Fig. 9 C, D). During dupilumab treatment, the patient’s atopic dermatitis improved, as evidenced by reductions in Scoring Atopic Dermatitis (SCORAD) and Eczema Area and Severity Index (EASI) scores (Fig. 9 A). To objectively assess the impact of dupilumab, we measured various serum biomarkers at baseline (t = 0), and after 30 and 150 days of treatment. Dupilumab significantly reduced the Th1 cytokine IFN-γ, although TNFα levels remained unchanged (Fig. 9B). Th2-related cytokines, including IL-13, IL-5 and IL-10, as well as IgE, also decreased over time, as anticipated (Fig. 9 C). Interestingly, serum IL-4 levels increased, which may reflect a compensatory response or serum accumulation due to IL-4 receptor blockade by dupilumab (Fig. 9 C). IL-17 A levels remained stable, with a slight increase observed during treatment (Fig. 9D). Evidence of remission from chronic general inflammation was observed through reduced serum levels of the pro-inflammatory cytokines IL-1β and IL-6 (Fig. 9E), as well as C-reactive protein (Fig. 9 F). Additionally, blood count-derived inflammatory markers have been shown to be useful for monitoring during dupilumab treatment [21]. Specifically, the platelet-to-lymphocyte ratio (PLR), monocyte-to-lymphocyte ratio (MLR), neutrophil-to-lymphocyte ratio (NLR), systemic inflammation response index (SIRI), and systemic immune-inflammation index (SII) all decreased during treatment with dupilumab in our patient, again indicating resolution of overall systemic inflammation. Notably, the percentage of circulating T_REGS_ increased from a baseline level of 0.96–2.18% (Fig. 9H, I), which may indicate a partial resolution of immune dysregulation induced by dupilumab. In summary, these findings suggest that dupilumab treatment in the patient with the *ACTB* variant effectively managed atopic dermatitis, while also reducing overall inflammation and potentially shifting immune responses by suppressing both Th1 and Th2 pathways, while preserving Th17-mediated immunity.

## Discussion

This study is the first to associate *ACTB* variants with T-cell immune dysregulation in BRWS1, providing mechanistic insights into cytoskeletal control of immune function and potential therapeutic interventions.

Actin dynamics play a crucial role in cellular development, activation and proliferation [18]. The macro-thrombocytopenia observed in our patient aligns with findings from a previous study, which identified impaired platelet development and maturation in six patients with various variants in exons 5 and 6 of *ACTB*, all presenting with syndromic thrombocytopenia [10]. Notably, our in-vitro results demonstrate impaired T-cell activation and proliferation. The decreased quantity of T_REGS_ contributes to the immune dysregulation seen in our patient, particularly manifested through asthma and atopic disorders. Among its pivotal functions, the T-cell cytoskeleton is involved in forming the immunological synapse by transporting various organelles to this site [22]. Additionally, the reorganization of supramolecular activation clusters (SMACs) involves moving distal (d)SMACs towards the immunological synapse, which is essential for positioning central SMACs (cSMACs). This process is vital for initiating TCR signaling [22].

Our study offers new insights into the cellular mechanisms underlying the *ACTB* variant. We demonstrate a dominant negative effect using confocal microscopy of HEK293T cells co-transfected with WT and p.Gln360ProfsTer4 plasmids, thus revealing that the presence of the mutant β-actin disrupts the normal peripheral distribution of WT β-actin. In addition, we found that the mutant β-actin is unstable with accelerated degradation within T cells, as demonstrated by immunoblotting studies performed with the addition of CHX, a protein translation inhibitor. Dominant-negative variants typically result in milder phenotypes than loss-of-function variants [23], which may contribute to patient survival, considering the essential roles of β-actin.

Rescue of T-cell activation by the addition of exogenous IL-2 in actinopathies was previously reported by our group and others in CARMIL2 deficiency [15, 24] and WAS protein knockout mouse model [25]. The compromised TCR signaling in T cells harboring the p.Gln360ProfsTer4 *ACTB* dominant negative variant leads to reduced IL-2 secretion, resulting in a lower local concentration of IL-2 at the immunological synapse. Previous studies have shown that IL-2’s paracrine activity in this synapse enhances T-cell activation, with a more effective signal transducer and activator of transcription (STAT)5 phosphorylation being one of the mechanisms involved [26]. In our study, the addition of exogenous IL-2 partially rescued cytokine production and T-cell proliferation in a dose-dependent manner, possibly by increasing the local IL-2 concentration at the immunological synapse, thus enhancing IL-2’s paracrine action. Low-dose IL-2 has been investigated as a potential treatment for T_REGS_ dysfunction in inflammatory and autoimmune disorders [27]. Additionally, due to concerns regarding IL-2’s adverse reactions, monoclonal antibodies that act as agonists for the IL-2 receptor are currently under investigation. One such antibody is rezpegaldesleukin, which has been recently shown to enhance T_REGS_ proliferation [28].

Another targeted treatment we explored in our study is dupilumab, making this the first report to describe its successful use in BRWS1. We demonstrate not only an improvement in type 2 inflammation but also a significant reduction in overall systemic inflammatory markers. Similar findings have been attributed to dupilumab treatment in previous reports [29]. Furthermore, we show that in our BRWS1 patient, dupilumab induces a shift from a Th2-dominated response to a Th17-skewed profile. This aligns with previous reports of dupilumab-induced psoriasis, a Th17-mediated disorder [30–32].

Over the past few years, the use of dupilumab in IEI has gained recognition, with increasing reports of its effective and safe treatment for conditions such as CARD11-associated atopy with predominant NF-κB signaling interference (CADINS) [33, 34], hyper-IgE syndrome induced by dominant negative variants in *STAT3* [35–39], as well as DOCK8 deficiency [40],WAS [41] and other IEIs [42, 43]. Our BRWS1 patient responded positively to dupilumab, as shown by the clinical and biological markers, highlighting its potential use for actinopathy-driven immunodeficiency.

The clinical evaluation of BRWS1 rarely involves immunologists, despite reports of recurrent sinopulmonary infections, as previously demonstrated in a series of 33 patients [7], along with prior evidence of neutrophil dysfunction [9]. To date, no in-depth analysis of T-cell functions has been conducted in these patients. Through our findings, we highlight the need for routine cellular and humoral immune assessments in these patients. This approach could improve patient care through targeted interventions, such as prophylactic antibiotics, IVIG and biological treatment.

A key limitation of our study is its reliance on a single BRWS1 patient. However, given the rarity of BRWS1, we have extensively validated our findings using a robust in-vitro transfection model to provide detailed mechanistic insights. Furthermore, the phenotypic rescue achieved through exogenous IL-2 and dupilumab treatment strengthens the validity of our results.

In conclusion, the novel dominant-negative ACTB variant, p.Gln360ProfsTer4, produces an unstable β-actin, leading to its rapid degradation in T cells and compromising effector functions. Targeted therapeutic approaches, such as IL-2 and dupilumab, warrant further investigation and may have broader applications in other actinopathies.

## Methods

### Study Design

This is an analysis of the medical records and laboratory immune workup of a single patient (P1). P1 was diagnosed and treated at the allergy and clinical immunology unit, Hadassah Medical Center, Jerusalem, Israel in the period of 2020–2023. Further in-depth phenotypic and mechanistic experiments were completed using an in-vitro model of transiently transfected HEK293T cell line.

### Exome Sequencing

Exome sequencing was performed using the Twist Human Core Exome Plus Kit (Twist Bioscience, San Francisco, CA, USA) on a NovaSeq 6000 sequencing machine (Illumina, San.

Diego, CA, USA). For each sample, paired-end reads (2 × 100 bp) were obtained and processed. The Illumina Dragen Bio-IT Platform version 3.9 was used to align reads to the human reference genome (hg38) based on the Smith-Waterman algorithm [44], as well as to call variants based on the GATK variant caller version 3.7 [45]. Additional variants were called by Freebayes version 1.2.0 [46]. Variant annotation was performed using KGG-Seq version 1.2 [47]. Further annotation and filtration steps were performed by in-house scripts using various additional datasets. Suspected pathogenic variants were validated through Sanger sequencing and family segregation studies.

### Lymphocyte Immune Phenotyping

Human PBMC were purified using density gradient medium centrifugation (Cat #: 07861, Lymphoprep™, Alere Technologies AS, Oslo, Norway). They were then stained using anti-CD3, CD4, CD8, CD19, CD20, CD45RA, CD45RO and CD56 antibodies (Beckman Coulter, California, USA). Flow cytometry was used to characterize the different lymphocyte subsets. Analysis of flow-cytometry readouts was done with FCS Express 6.0 (De-Novo software, California, USA) and Kaluza software (Beckman Coulter, California, USA).

### Regulatory T-Cell Counting and T-Cell Receptor Vβ Repertoire

Methodological details regarding T_REGS_ quantification and TCR Vβ repertoire were previously described [48]. PBMC of P1 and healthy controls (HC) were surface stained for CD4 and CD25 and then fixated and perambulated using FOXP3 staining buffer set, according to the manufacturer’s protocol (Invitrogen, eBioscience, California, USA). This was followed by intra-nuclear staining for FOXP3^+^, using anti-FOXP3 antibody (Miltenyi Biotec, Auburn, California, USA). In addition, TCR Vβ repertoire was determined by flow-cytometry and was completed according to the manufacturer’s manual (Beta Mark TCR Vβ Repertoire Kit; Beckman Coulter, California, USA).

### Cryo-Modeling and Immunoblotting

A cryo-electron microscopy model of β-actin in the patient and a healthy control was compiled using the PyMOL Molecular Graphics System, Version 3.0, Schrödinger, LLC. (protein data bank: 5JLH [49].

Protein lysates were extracted from the PBMC of P1 and HC using a radioimmunoprecipitation assay (RIPA) buffer (Cat # 89900, Thermo Fisher Scientific, Massachusetts, USA) containing a protease inhibitor (Cat # 78429, Thermo Fisher Scientific, Massachusetts, USA). To accurately quantify the amount of protein in each lysate and confirm that the total protein amount is comparable between P1 and HC, we used the Bradford protein assay before running the assay (Cat # 5000006, Bio-Rad, US). Protein lysates were loaded to a nitrocellulose membrane and thereafter were subjected to blocking with 5% skimmed milk for 1 h at room temperature. Anti-β-actin antibody (Cat # A5441, Sigma, Missouri, USA) [13] was then utilized to detect the β-actin band. The membrane loaded with proteins was then incubated with the anti-β-actin antibody overnight at 4^o^C. Following readout of the β-actin band, the membrane was incubated with a stripping buffer (Cat # 21059, Thermo Fisher Scientific, Massachusetts, USA) for 15 min at room temperature. Finally, anti-Glyceraldehyde 3-phosphate dehydrogenase (Cat # CB1001, Sigma, Missouri, USA) antibody was used as a loading control.

### Analysis of T-Cell Proliferation Capacity

Freshly isolated PBMC from P1 and HC were marked with CTV (according to the manufacturer instructions (Cat #. C34571, ThermoFisher, Massachusetts, USA). In addition, IL-2 receptor α subunit (CD25; Cat #. 302609, Biolegend, California, USA) was used as a marker of T-cell activation. PBMC were then seeded at 3 × 10^6^/200 µl into a 96-well flat bottom plate and activated with T-Cell TransAct reagent (Cat #. 130-111-160, Miltenyi Biotec, Germany) and human interleukin (IL)−2 (0, 100 and 400 U/ml; Proleukin^®^; Iovance Biotherapeutics, California, USA). Active T-cell rosettes were examined using light microscopy following 48 h of activation. Following 120 h of activation, cells were harvested and readout of the CTV and CD25 signals of CD4^+^ T cells were completed using flow-cytometry. Assessing CD4^+^ T-cell proliferation capacity was then done with calculation of the proliferation indices for each subject. In addition, at this time point, supernatants were collected from each well and kept at −80°C for further quantification of cytokines that were secreted during cellular activation.

### Quantification of Cytokines in the Sera and T-Cell Supernatants Following Activation

Sera from P1 and HC were separated from whole blood by centrifugation (1000 g, 20 min, 4 °C) and stored at −80°C until use. Baseline cytokines in the sera and cell supernatants following 120 h of In-vitro activation were quantified by enzyme-linked immunosorbent assay (ELISA). The average of samples taken from five age-matched healthy controls and literature-based normal ranges [50] were used for comparisons. Serum levels Investigated cytokines consisted of interferon (IFN)-γ (Cat #. 430104), interleukin (IL)−13 (Cat #. 435207), IL-4 (Cat #. 430304), IL-17 (Cat #. 433914) and IL-5 (Cat #. 430404); Biolegend, California, USA. Additionally, tumor necrosis factor (TNF)-α, IL-1β, IL-6, IL-10 and soluble IL-2 receptor α (sIL-2Rα) from P1’s and HC’s sera were measured with Immulite^®^ 1000 immunoassay system (Siemens Healthineers, Germany). All assays were conducted according to the manufacturer’s protocols.

### Assessment of Reactive Oxygen Species Production by Neutrophils

#### Human Neutrophil Purification

Freshly drawn heparinized blood (20U/ml) was combined with an equivalent volume of Dextran 500 (3% in saline) and allowed to incubate for 30 min at room temperature. Subsequently, the leukocyte rich supernatant was carefully layered onto Histopaque 1077 (Sigma) and subjected to centrifugation. High-density neutrophils were selectively collected in the pellet fraction. To eliminate contaminating erythrocytes, neutrophils were suspended in 10 ml of 0.2% NaCl for 30 s, followed by restoration of isotonicity through the addition of 10 ml of 1.6% NaCl. The neutrophils were then subjected to one wash in Hank’s Balanced Salt Solution (HBSS) devoid of phenol red.

#### Reactive Oxygen Species Production Analysis

Ten thousand isolated neutrophils in HBSS were seeded into a white 96-flat-bottom well plate. Subsequently, a 20 µl solution containing luminol (500 µM) and horseradish peroxidase (40 U/ml) in HBSS, was introduced into each well. Where indicated, the neutrophils were treated with 10 nM phorbol 12-myristate 13-acetate (PMA). Chemiluminescence was monitored for duration of 60 min using the InfiniteF200Pro system (TECAN, Männedorf, Switzerland).

In addition, intra-cellular ROS were analyzed using dihydrorhodamine**-**1,2,3 (DHR) oxidation assay according to the manufacturer’s protocol (FagoFlowEx Kit, exbio ED7042, Czechia), as previously detailed by Vowells et al. [51].

### Introduction of the p.Gln360ProfsTer4 variant into HEK293T cells via transfection

#### Cell Culture

HEK293T cells were cultured in Dulbecco’s Modified Eagle Medium (DMEM) supplemented with 10% heat-inactivated fetal bovine serum, 100 U/mL penicillin-streptomycin, and 2 mM glutamine at 37 °C under humidified air containing 5% CO₂.

#### Plasmids

HA-Tagged WT *ACTB* or FLAG-Tagged p.Gln360ProfsTer4 variant *ACTB* were cloned into expression plasmid: pRP[Exp]-Puro-EF1A.

#### Transient Transfection Protocol

HEK293T cells were seeded at 50% confluency and cultured for one day before transfection to reach 70–80% confluency (5 million cells per 10 cm dish in 10 mL of medium) on the day of transfection. The medium was changed before transfection, which was performed using PEI Max transfection-grade reagent (Polysciences, Inc., Cat #24765) at a stock concentration of 3 µg/µL and OptiMEM (Gibco, Cat #31985047). For each reaction, 0.7 µL of PEI was used per 1 µg of DNA. After 14 h of transfection with 12 µg of actin constructs, the medium was removed and replaced with fresh DMEM. Seventy-two hours after transfection, cells were analyzed by flow cytometry, microscopy, and immunoblotting.

#### Flow Cytometry and Intracellular Staining

Cells were then harvested and centrifuged at 300 g for 5 min. The supernatant was removed, and the cell pellets were re-suspended in 100% cold methanol and incubated for 20 min at −20°C. After incubation, the cells were centrifuged at 300 g for 5 min. The methanol was removed, and the cell pellets were re-suspended in anti-FLAG (Biolegend, Cat. #637308) and anti-HA (Biolegend, Cat #901517) antibodies diluted 1:1000 in FACS buffer (PBS supplemented with 2% FBS and 1 mM EDTA) for 30 min at 4 °C.

### Confocal Microscopy

#### Assessing Actin Filaments in Activated T Cells

Peripheral blood mononuclear cells (PBMCs) derived from P1 and three healthy controls (HC) were activated in a 96-well flat-bottom plate using 400 ng/ml of suspended anti-CD3/anti-CD28 activating antibodies (BioLegend, Cat #317326 and #302923). After 24 h, the activated T cells were stained with Phalloidin-iFluor 647 Reagent (Abcam, Cat #ab176759) and anti-CD3, FITC (eBioscience, Cat #9011-0036-120). Cells were fixed with 2% paraformaldehyde (PFA) for 30 min at 4 °C and then stained in a blocking solution containing 3% bovine serum albumin (BSA), 1% normal goat serum (NGS), 0.03%Tween-20, and 0.1% Triton X-100 for 2 h at room temperature. Subsequently, the cells were washed three times with PBST, stained with DAPI (Thermo Fisher Scientific, Cat #D1306), and mounted using ibidi Mounting Medium (ibidi, Cat #50001).

#### Evaluation of Immunological Synapse Formation Upon Activation

Isolated T cells were activated for 16 h using Dynabeads™ Human T-Expander CD3/CD28 (Thermo Fisher, catalog number: 11141D) according to the manufacturer’s instructions. The cells were then adhered to Poly-D-Lysine Hydrobromide (Sigma, catalog number: A-003-M)-coated µ-Slide 8 Well Glass Bottom (ibidi, catalog number: 80827). After adherence, the cells were fixed with 2% paraformaldehyde (PFA) for 30 min at 4 °C, permeabilized with 0.1% Triton X-100 in PBS, and stained for IL-2 (PE, BioLegend, catalog number: 500307), CD3 (Alexa Fluor 488, BioLegend, catalog number: 300415), and Phalloidin-iFluor 647 Reagent (Abcam, catalog number: ab176759) for 3 h at room temperature in a blocking buffer containing 3% bovine serum albumin (BSA), 1% goat serum, and 0.03% Tween-20. Subsequently, the samples were stained with DAPI (Thermo Fisher Scientific, catalog number: D1306) and mounted using ProLong Gold Antifade Mountant (Thermo Fisher Scientific, catalog number: P10144). Images were acquired using a Zeiss LSM 980 Confocal Microscope with Airyscan 2.

#### Co-localization Analysis of Transfected HEK293T Cells Using Phalloidin and Epitope-Specific Antibodies

Transfected HEK293T Cells were cultured overnight in µ-Slide 8 Well high Glass Bottom slides (ibidi, Cat #: 80807) with DMEM medium to allow for cell adherence. The cells were then carefully washed twice with PBS and fixed with 4% PFA for 30 min at 4 °C. The cells were blocked and stained in a solution containing 3% BSA, 0.03% Tween-20, and 0.1% Triton X-100, along with Phalloidin-iFluor 488 Reagent (Abcam, Cat #ab176753) and antibodies against HA (PE) and FLAG (APC) for 2 h at room temperature. Subsequently, the cells were carefully washed with PBST, stained with DAPI (Thermo Fisher Scientific, D1306), and mounted using ibidi Mounting Medium (ibidi, Cat # 50001). Images were taken using a ZEISS LSM 980 with Airyscan. Method for calculation of co-localization was previously detailed [52]. 

## Electronic Supplementary Material

Below is the link to the electronic supplementary material.

Supplementary Material 1

## Data Availability

Data is available upon request from the corresponding author.

## Declarations

### Study Approval

The study was approved by Hadassah’s institutional review board (IRB#: 0337-24-HMO). Written informed consent for participation in the study was obtained from the patient.
