# Supplementary material for: β-Actin Deficiency in Baraitser-Winter Syndrome Type 1 Disrupts T-Cell Function and Immune Regulation: Implications for Targeted Therapy in Actinopathies
Source: J Clin Immunol. 2025 Aug 1;45(1):120. doi: 10.1007/s10875-025-01906-x (PMC12316732; doi:10.1007/s10875-025-01906-x)
Supplement: Supplementary file 1 — Supplementary Material 1 [file 10875_2025_1906_MOESM1_ESM.docx]

**Supplementary data**

**β-Actin Deficiency in Baraitser-Winter Syndrome Type 1 Disrupts T-Cell Function and Immune Regulation: Implications for Targeted Therapy in Actinopathies**

Zahala Bar-on, Or Reuven, Atar Lev, Amos J. Simon, Wajeeh Salaymeh, Alit Shalom, Raz Somech, Ortal Barel, Sigal Porges, Elisheva Javasky, Vered Molho-Pessach, Zvi Granot, Dan Bijaoui, Tzahi Neuman, Yuval Tal, Michal Baniyash, Michael Berger, Oded Shamriz

**This document contains Supplementary Figures 1-6.**

| 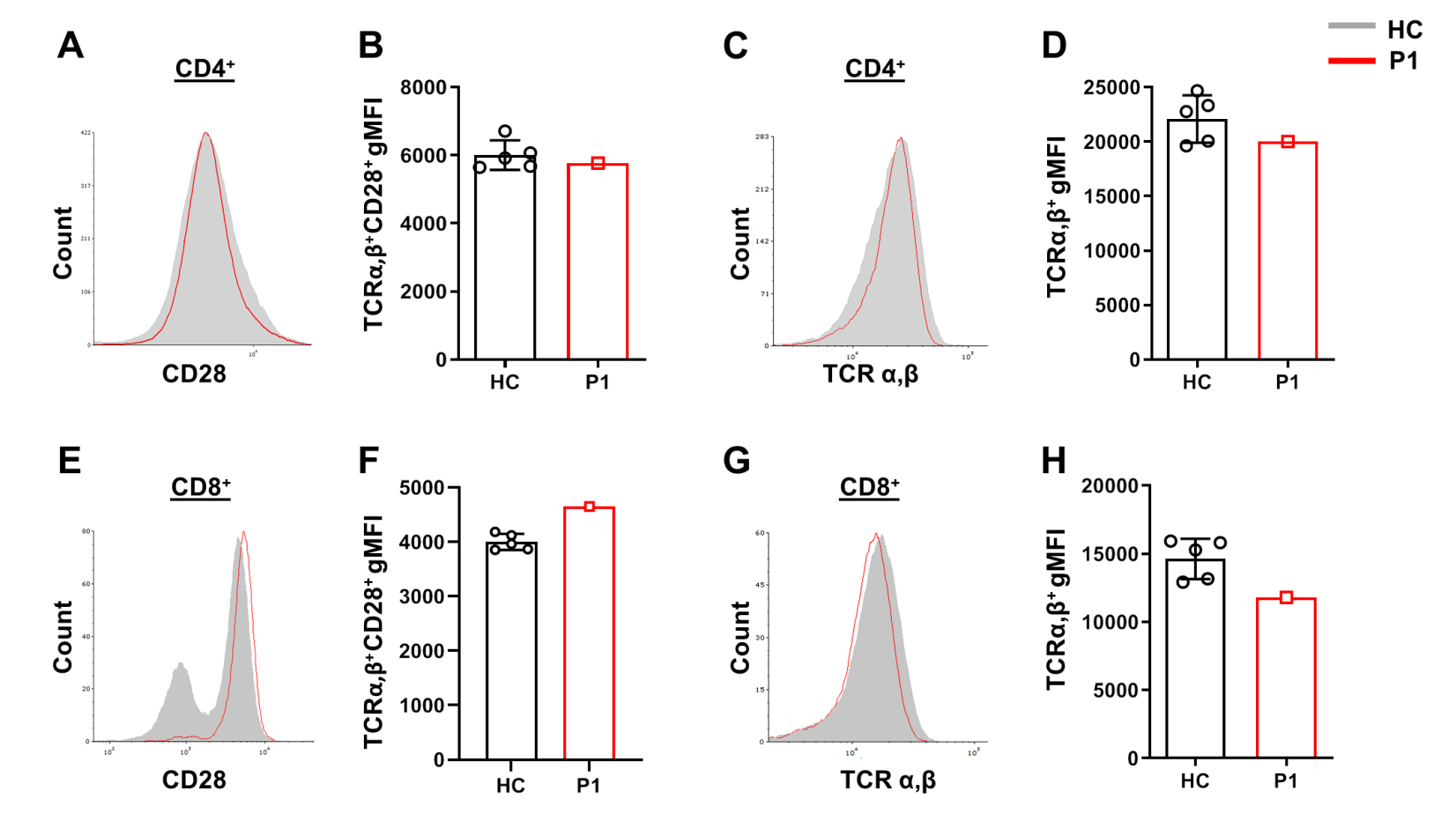 |
| --- |

**Supplementary Figure 1. Flow cytometric analysis of TCRα, β and CD28 surface expression in the patient and healthy controls.** PBMCs were purified and stained with antibodies against TCRα, β and CD28 and compared to healthy controls. (A-D) present histograms of mean fluorescence indices of TCRα, β and CD28 upon the surface of CD4^+^ T cells. (E-H) Same as above for CD8^+^ T cells**.**

| 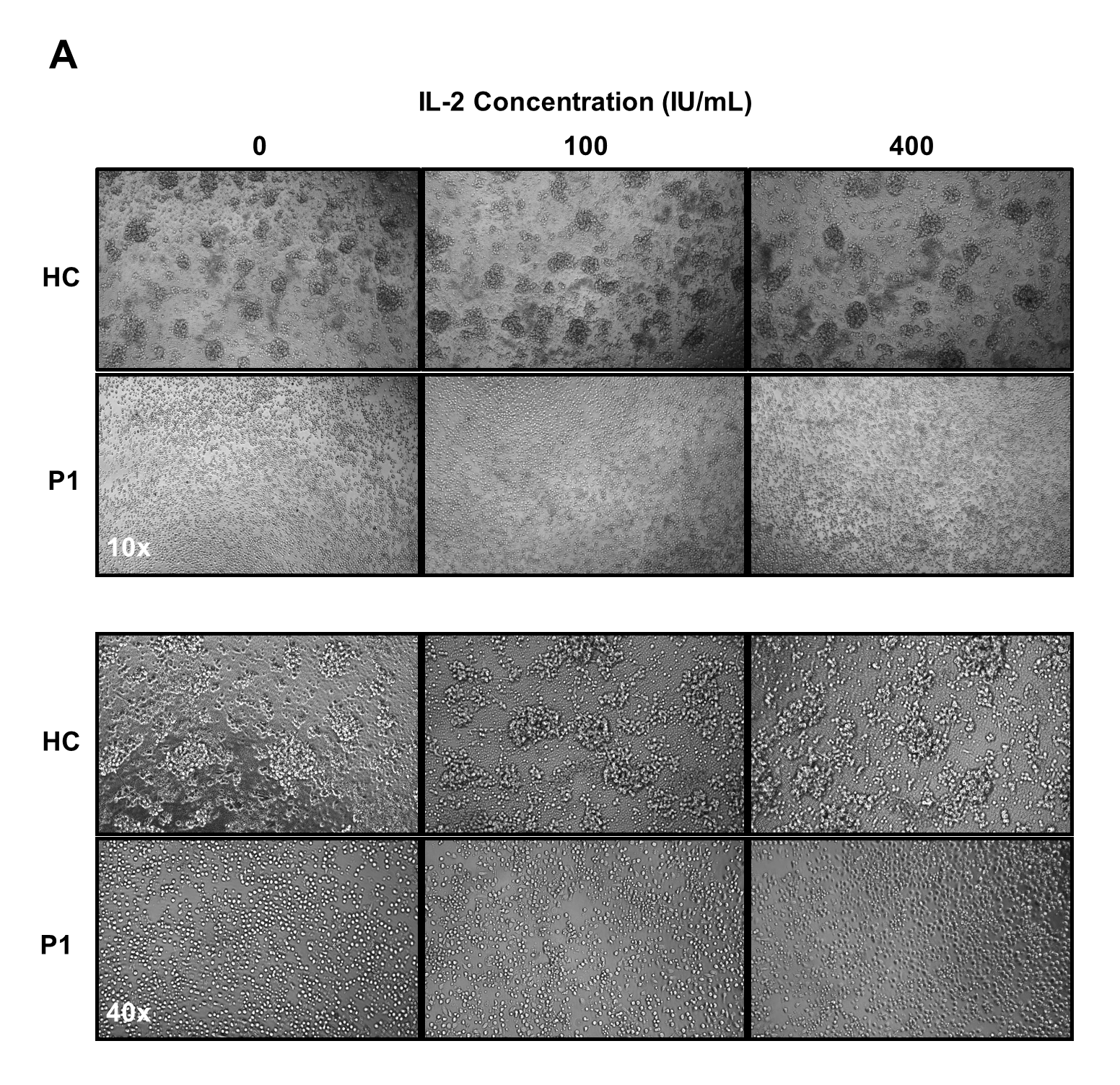 |
| --- |

**Supplementary Figure 2. Active T-cell rosettes following activation in the patient and a healthy control**

(A-B) PBMCs were purified and activated in vitro for 48 hours using anti-CD3 and anti-CD28-coated beads. T-cell rosettes from the patient and a healthy control were then visualized using light microscopy.

| 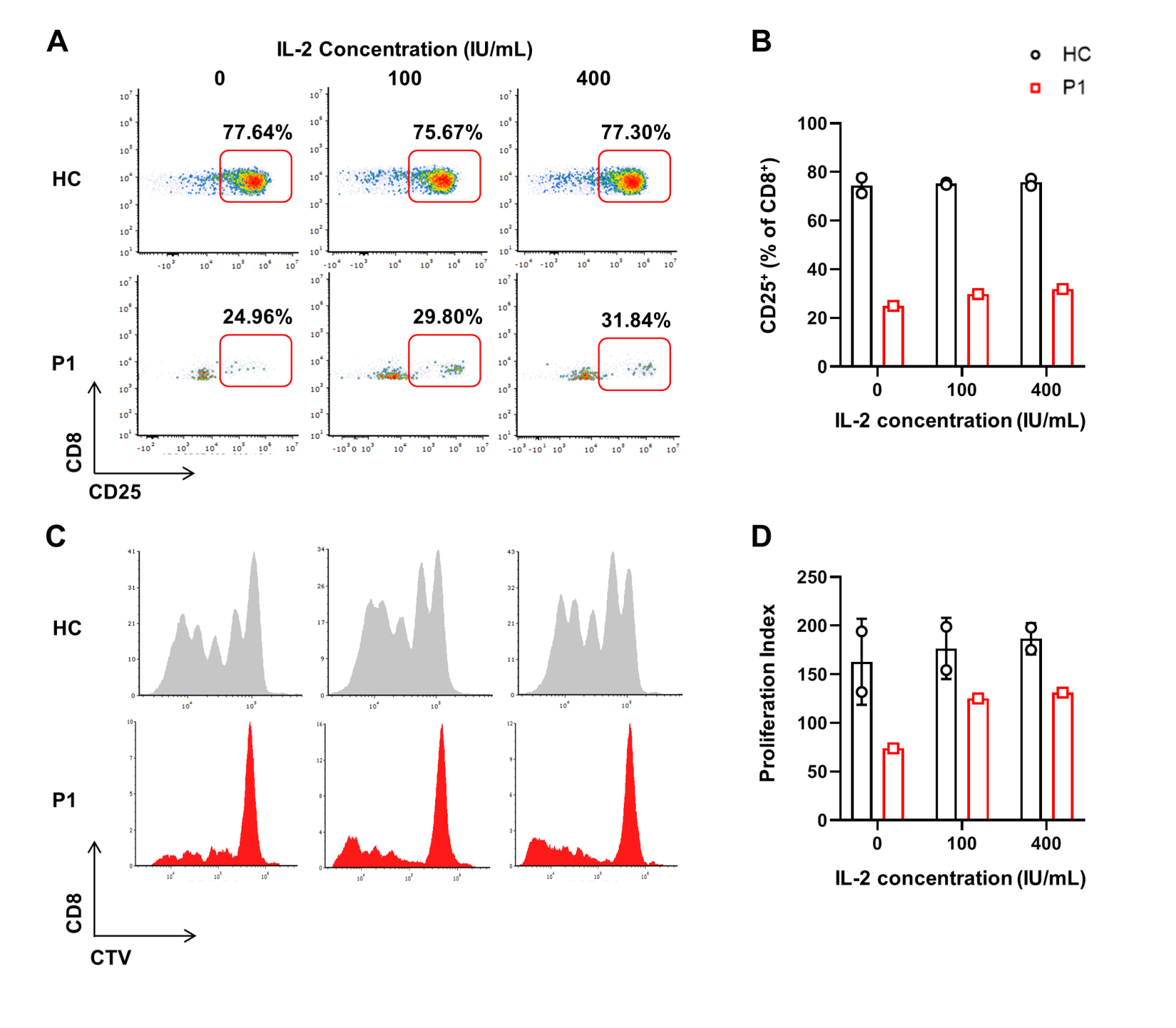 | |
| --- | --- |
|  |  |
| **Supplementary Figure 3. Analysis of CD8^+^ T-cell activation, cytokine secretion, and proliferation in the presence of exogenous IL-2.** CD8^+^ T cells derived from the patient and healthy controls were activated using anti-CD3/CD28 coated beads with addition of exogenous IL-2 in concentrations of 0, 100 and 400 IU/mL, as detailed in Fig 7. (A-B) Left, density plot demonstrating CD8^+^CD25^+^ surface expression, right, summary in bar graph. (C-D) T-cell proliferation capacity presented as histogram. (C) A bar graph summering proliferation indices in the patient and healthy controls. |  |

|  |
| --- |
| \| **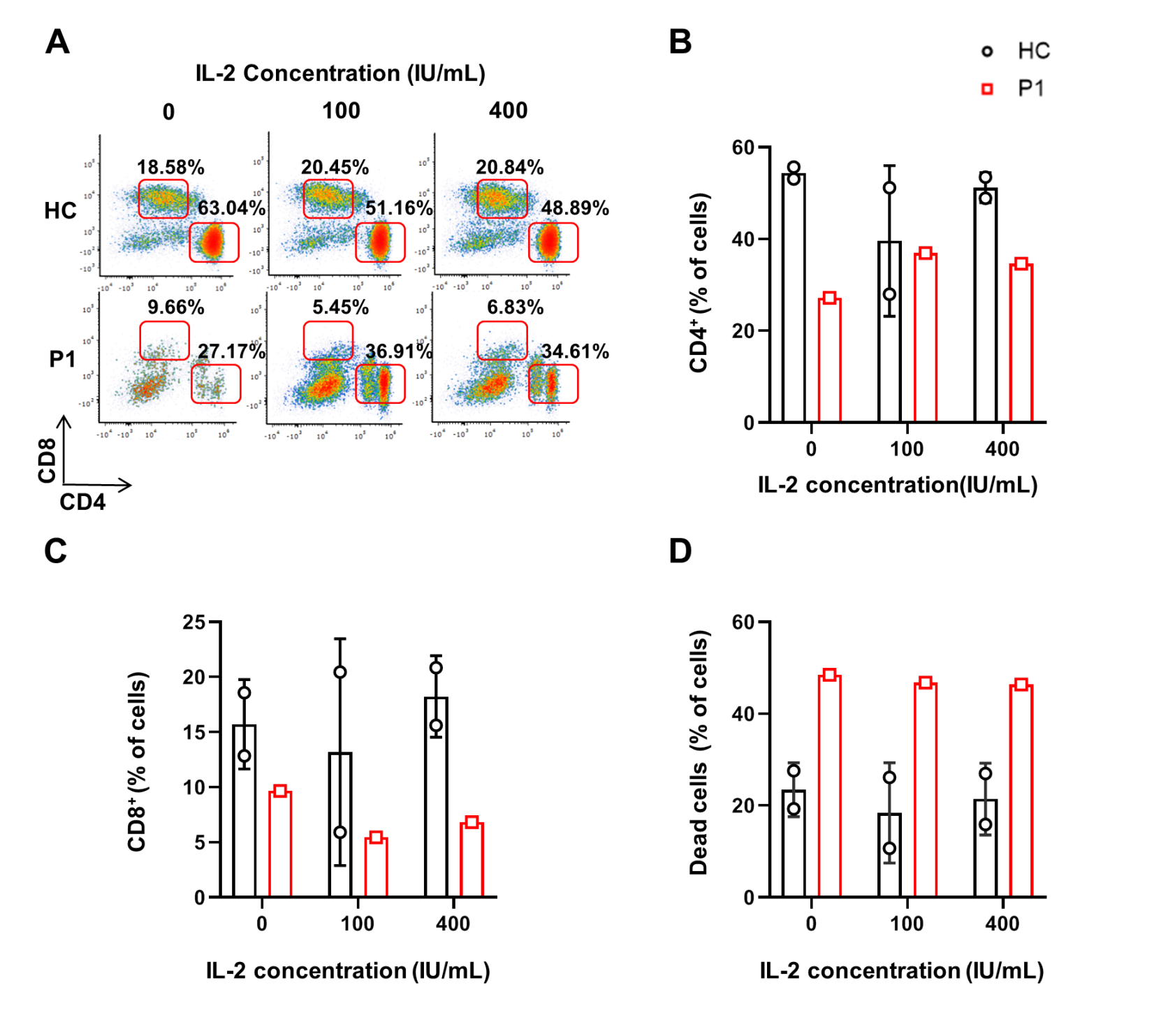** \| \| --- \|   **Supplementary Figure 4. Analysis of activation-induced cell death in the patient and healthy controls.**  (A-C) CD4^+^ and CD8^+^ T cells were activated as explained before. Following activation CD4+, CD8+ T-cell subsets were quantified using flow cytometry. (D) Activation-induced cell death (AICD) was evaluated by quantifying dead cells following activation in the patient and healthy controls by using flow-cytometry. |

| 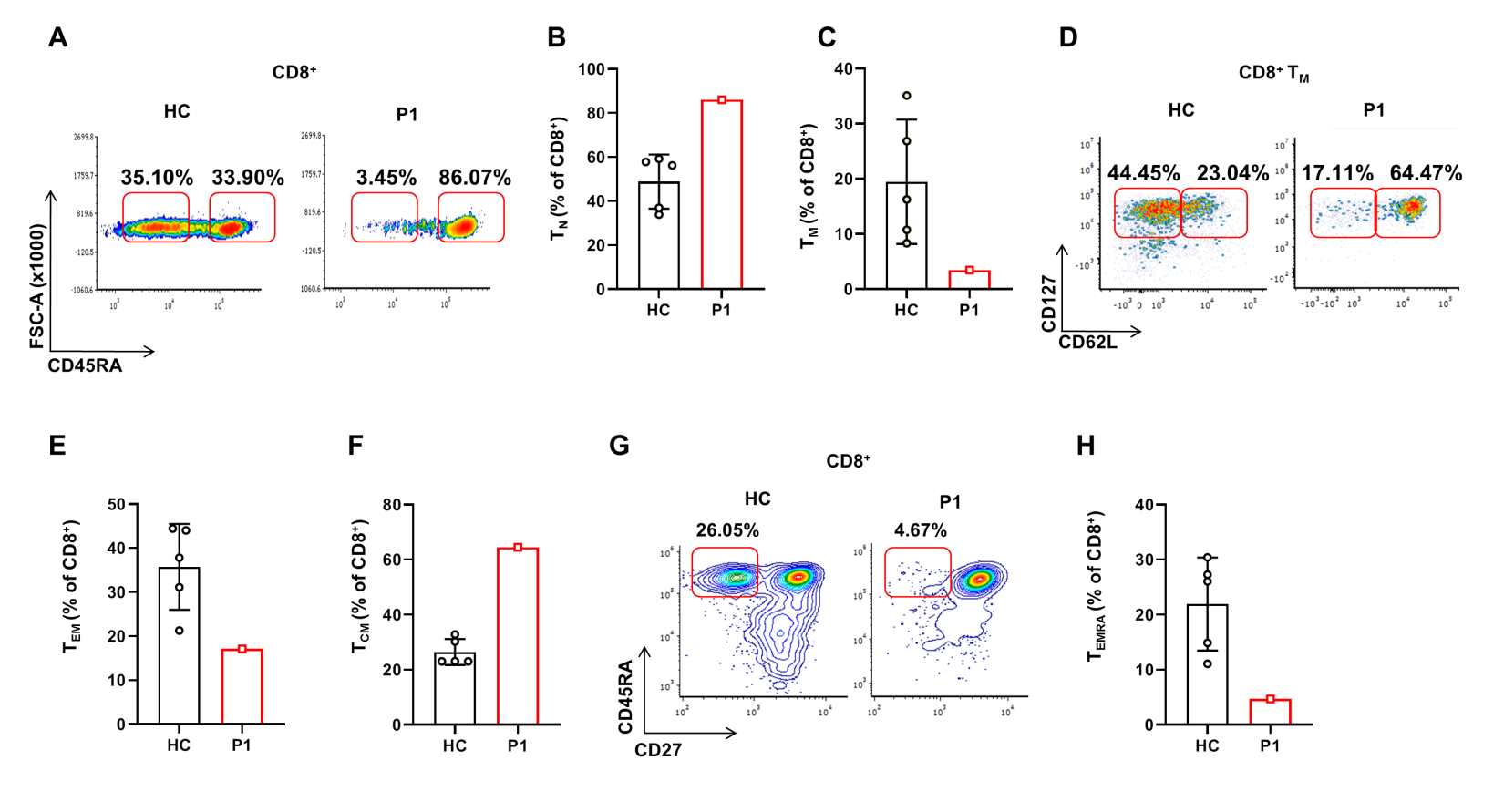 |
| --- |

**Supplementary Fig 5. Analysis of CD8^+^ T-cell subsets.** (A-F) The patient's naïve, total memory, effector memory and central memory CD8^+^ T cells were quantified using flow-cytometry and compared to healthy controls. (G-H) Same as above, for CD8^+^ T_EMRA_ cells.

|  |
| --- |
| \| **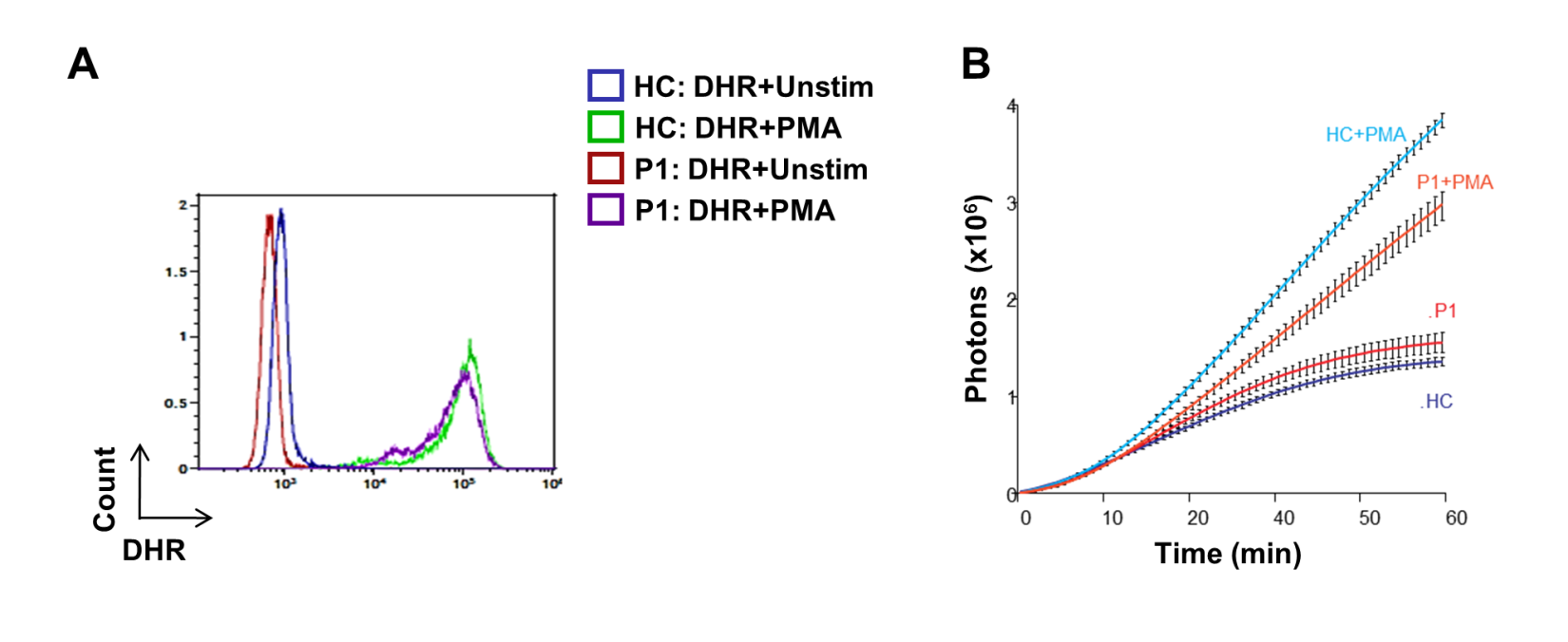** \| \| --- \|   **Supplementary Figure 6. Reactive oxygen species production in the patient and a healthy control**. (A) Intra-cellular reactive oxygen species (ROS) production in the patient and healthy control were analyzed using dihydrorhodamine‐1,2,3 (DHR) oxidation assay.(B) Neutrophils derived from the patient and a healthy control were treated with 10 nM phorbol 12-myristate 13-acetate (PMA). Chemiluminescence was then monitored for duration of 60 minutes using the InfiniteF200Pro system.   \| Vβ clone  (% of TCR expression) \| Patient \| Healthy controls \| \| \| --- \| --- \| --- \| --- \| \| Mean \| SD \| \| VB 1 \| 2.88 \| 3.53 \| 1.35 \| \| VB 2 \| 9.66 \| 8.3 \| 2.46 \| \| VB 3 \| 10.15 \| 4.68 \| 3.13 \| \| VB 4 \| 2.71 \| 1.91 \| 0.48 \| \| VB 5.1 \| 6.5 \| 5.45 \| 1.6 \| \| VB 5.2 \| 1.05 \| 1.33 \| 0.52 \| \| VB 5.3 \| 0.88 \| 1.08 \| 0.42 \| \| VB 7.1 \| 3.12 \| 2.56 \| 2.08 \| \| VB 7.2 \| 0.59 \| 1.47 \| 1.03 \| \| VB 8 \| 3.14 \| 4.68 \| 2.94 \| \| VB 9 \| 3.23 \| 3.13 \| 1.15 \| \| VB 11 \| 0.98 \| 1.04 \| 0.62 \| \| VB 12 \| 1.54 \| 1.66 \| 0.54 \| \| VB 13.1 \| 3.81 \| 3.83 \| 1.06 \| \| VB 13.2 \| 1.4 \| 2.8 \| 1.23 \| \| VB 13.6 \| 2.06 \| 1.86 \| 0.96 \| \| VB 14 \| 4.01 \| 3.49 \| 1.36 \| \| VB 16 \| 0.64 \| 0.92 \| 0.29 \| \| VB 17 \| 4.87 \| 5.15 \| 1.28 \| \| VB 18 \| 0.93 \| 1.49 \| 0.74 \| \| VB 20 \| 1.5 \| 2.52 \| 1.38 \| \| VB 21.3 \| 2.27 \| 2.38 \| 0.72 \| \| VB 22 \| 4.99 \| 3.84 \| 1.17 \| \| VB 23 \| 0.97 \| 0.85 \| 0.65 \|   **Supplementary Table 1: T-Cell Receptor Vβ Repertoire of the Patient and Healthy Controls** Data represent the CD3^+^ T-cell receptor (TCR) Vβ repertoire analyzed by flow cytometry. Clone distribution is shown as the percentage of TCR expression in the patient and the mean (±SD) of 85 healthy controls.  *.* |
